# Structural Insight into the Catalytic Mechanisms of an L‐Sorbosone Dehydrogenase

**DOI:** 10.1002/advs.202301955

**Published:** 2023-09-07

**Authors:** Dong Li, Zhiwei Deng, Xiaodong Hou, Zhijie Qin, Xinglong Wang, Dejing Yin, Yue Chen, Yijian Rao, Jian Chen, Jingwen Zhou

**Affiliations:** ^1^ Engineering Research Center of Ministry of Education on Food Synthetic Biotechnology Jiangnan University 1800 Lihu Road Wuxi Jiangsu 214122 China; ^2^ Science Center for Future Foods Jiangnan University 1800 Lihu Road Wuxi Jiangsu 214122 China; ^3^ Key Laboratory of Industrial Biotechnology Ministry of Education and School of Biotechnology Jiangnan University 1800 Lihu Road Wuxi Jiangsu 214122 China; ^4^ Jiangsu Province Engineering Research Center of Food Synthetic Biotechnology Jiangnan University Wuxi 214122 China

**Keywords:** 2‐keto‐L‐gulonic acid, *Gluconobacter oxydans*, redox control, substrate pocket, Vitamin C

## Abstract

L‐Sorbosone dehydrogenase (SNDH) is a key enzyme involved in the biosynthesis of 2‐keto‐L‐gulonic acid , which is a direct precursor for the industrial scale production of vitamin C. Elucidating the structure and the catalytic mechanism is essential for improving SNDH performance. By solving the crystal structures of SNDH from *Gluconobacter oxydans* WSH‐004, a reversible disulfide bond between Cys295 and the catalytic Cys296 residues is discovered. It allowed SNDH to switch between oxidation and reduction states, resulting in opening or closing the substrate pocket. Moreover, the Cys296 is found to affect the NADP^+^ binding pose with SNDH. Combining the in vitro biochemical and site‐directed mutagenesis studies, the redox‐based dynamic regulation and the catalytic mechanisms of SNDH are proposed. Moreover, the mutants with enhanced activity are obtained by extending substrate channels. This study not only elucidates the physiological control mechanism of the dehydrogenase, but also provides a theoretical basis for engineering similar enzymes.

## Graphical abstract

Based on the analysis of SNDH structure, this study proposes a redox mechanism of SNDH, which allows the substrate pocket to be opened or closed, thereby affecting its activity. In addition, through the analysis of substrate channel of SNDH, the bottom tunnel bottleneck area is identified, and by releasing it, a mutant with significantly enhanced activity is obtained.

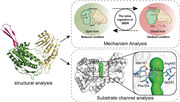

## 1 Introduction

Vitamin C is a trace element that is required by humans. It cannot be synthesized by human beings and can only be obtained from external sources.^[^ ^1^, ^2^ ^]^ Active oxygen in the body can be cleared by vitamin C to avoid cell damage.^[^ ^3^ ^]^ Vitamin C is not only involved in the physiological regulation process in the cell, but also a cofactor of biosynthesis and gene regulation enzyme families, such as monooxygenase, dioxygenase enzymes, and hydroxylase.^[^ ^4^, ^5^, ^6^ ^]^ Studies have shown that some critically ill patients will have symptoms of vitamin C deficiency, which can be relieved by high‐dose vitamin C supplementation.^[^ ^7^ ^]^ At present, vitamin C has been widely used in food, beverage, cosmetics, and feed.

**Figure 1:**
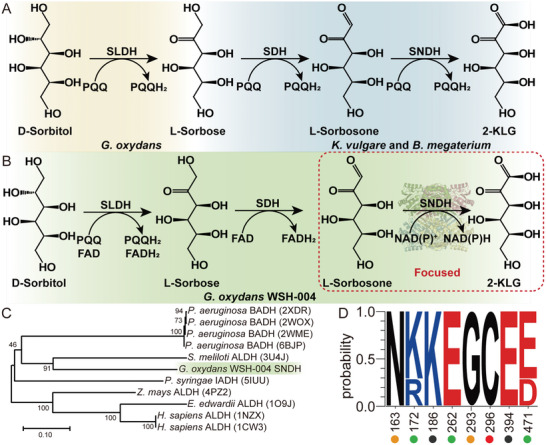
The role of SNDH in 2‐KLG biosynthesis and its sequence analysis. A)Schematic diagram of 2‐KLG production by the classical two‐step fermentation route. The yellow part represents the fermentation process of *Gluconobacter oxydans*, and the blue part represents the fermentation process of *Ketogulonicigenium vulgare* and *Bacillus sp*. B) The metabolic pathway of one step route of *G. oxydans* WSH‐004 to synthesize 2‐KLG. C) Phylogenetic tree of SNDH and its similar structures. D) Multiple sequence alignment of the key residues of SNDH and its similar structures. the catalytic residue, the proton transfer residues, the cofactor coordination residues, and the auxiliary residues are marked by red circle, green circles, black circles, and yellow circles, respectively.

2‐Keto‐L‐gulonic acid (2‐KLG) is a direct precursor for industrial vitamin C production.^[^ ^8^, ^9^ ^]^ The current industrial production method of vitamin C is the classical two‐step fermentation route (**Figure** **1**A).^[^ ^9^, ^10^ ^]^ The classical two‐step fermentation route method requires three bacteria (*Gluconobacter oxydans*, *Ketogulonicigenium vulgare*, and *Bacillus* sp.), two sterilization processes, and one extra material transfer, which make the process complicated and costly. Several attempts have been made to develop a one‐step fermentation method to produce 2‐KLG.^[^ ^11^, ^12^ ^]^ However, compared with the classical two‐step fermentation route, these methods are still noncompetitive.^[^ ^13^, ^14^ ^]^ In a previous report, the strain *G. oxydans* WSH‐004, which can directly convert D‐sorbitol into 2‐KLG, was obtained by a high‐throughput screening procedure based on 2‐KLG reductase,^[^ ^14^ ^]^ In *G. oxydans* WSH‐004, D‐sorbitol is converted to L‐sorbose through PQQ‐dependent sorbitol dehydrogenase (SLDH) or/and flavin adenine dinucleotide (FAD)‐dependent SLDH. Then, the FAD‐dependent sorbose dehydrogenase (SDH) converts L‐sorbose to L‐sorbosone. Finally, L‐sorbosone is further converted into 2‐KLG by a NAD(P)^+^‐dependent L‐sorbosone dehydrogenase (SNDH) (Figure 1B).^[^ ^2^, ^14^ ^]^ A previous study has shown that heterologous overexpression of FAD dependent SDH can produce a small amount of 2‐KLG, while co‐expression with NAD(P)^+^‐dependent SNDH can greatly increase the output of 2‐KLG.^[^ ^2^ ^]^ Therefore, elucidating the structure and the catalytic mechanism is essential for improving SNDH performance and the efficiency of 2‐KLG production.

Sequence analysis showed that the SNDH of *G. oxydans* WSH‐004 belongs to the aldehyde dehydrogenase (ALDH) superfamily (ALDH‐SF). The ALDH uses NAD(P)^+^ as a cofactor, widely exists in the six kingdoms of life, and plays a key role in the metabolism of toxic aldehydes and ketones in organisms.^[^ ^15^, ^16^ ^]^ Their overall structure is generally similar, but they have highly diverse catalytic activities.^[^ ^17^ ^]^ The ALDHs are usually homodimers or homotetramers.^[^ ^18^, ^19^ ^]^ The nucleophilic residue Cys and the catalytic base Glu are conserved catalytic amino acids in the ALDH active center, which can bind and release cofactors and substrates by changing the conformation during the catalysis process.^[^ ^20^, ^21^ ^]^ Interestingly, the substrate channels in the ALDH subfamily are very different, giving them high substrate diversity.^[^ ^20^, ^22^ ^]^ Some studies have also found a physically linked state of ALDH and ADH domains that can form biologically active higher‐order spirosome structures.^[^ ^23^ ^]^ At present, there is no report on the structure of the SNDH subfamily, and its catalytic mechanism and the substrate‐specific structural basis are still unknown. These factors limit the rational engineering of SNDHs.

The purpose of this research is to understand the catalytic mechanism of SNDH from *G. oxydans* WSH‐004 and conduct a semi‐reasonable design to improve the catalytic activity of SNDH. The crystal structural analysis found that the SNDH could exist in two different catalytic loop conformations, i.e., the oxidizing SNDH (O‐SNDH) with a closed substrate pocket and the reducing SNDH (R‐SNDH) with an open substrate pocket. To our knowledge, this phenomenon is the first report in microbial‐derived ALDHs. In addition, it was also found that the catalytic residue Cys296 can affect the binding pose of cofactors. Combined with previous reports and in vitro experiments, a dynamic regulation mechanism of redox environment dependence regulating the opening and closing of SNDH substrate pocket and the catalytic mechanism were proposed, respectively. Finally, the solved SNDH structure was used as the basis for engineering the substrate channel of SNDH to obtain mutants with enhanced activity. This work expands the current understanding of bacterial ketone dehydrogenases and lays a theoretical foundation for the use of ketone dehydrogenase and the industrial innovation of vitamin C production.

## 2 Results

### 2.1 SNDH is a Member of the ALDH Family

SNDH from *G. oxydans* WSH‐004 belongs to the ALDH‐SNDH subfamily in ALDH‐SF and has 504 amino acid residues. According to the sequence alignment (Table S1, Supporting Information) and phylogenetic analysis (Figure 1C), structure solved with the highest identity to SNDH is the ALDH from *Sinorhizobium meliloti* (PDB code: 3U4J), which has a coverage rate of 93% and a similarity of 46%. Multiple sequence alignment reveals that the residues related to catalytic reactions in the ALDH‐SF are highly conserved, including the catalytic residues Cys296, the proton transfer residues Arg172, Glu262 and Glu471, the cofactor coordination residues Lys186 and Glu394, and two auxiliary residues Asn163 and Gly293 (Figure 1D).^[^ ^24^, ^25^, ^26^ ^]^

SNDH with a C‐terminal His‐tag was expressed in *Escherichia coli* and purified. SDS‐PAGE analysis results showed that the molecular weight of SNDH is consistent with the theoretical size (54.4 kDa). Size‐exclusion chromatography data show that SNDH may be a homotetramer at the active state (Figure S1, Supporting Information). The enzymatic properties of SNDH were determined using L‐sorbosone as a substrate. According to cofactor tests, SNDH can use both NADP^+^ and NAD^+^. The catalytic rate with NADP^+^ is slightly better than that of NAD^+^ (Figure S2A, Supporting Information). High concentrations of NADP^+^ and NAD^+^ can inhibit the activity of SNDH to a certain extent. The optimum reaction temperature and pH for the SNDH are 50 °C (Figure S2B, Supporting Information) and 10.5 (Figure S2C, Supporting Information), respectively. Thermal stability assays showed that the SNDH is sensitive to temperatures higher than 40 °C, and has thermal stability at 30 °C (Figure S2D, Supporting Information). Among the common metal ions, Mg^2+^ can promote the activity, whereas Cu^2+^ has a substantial inhibitory effect (Figure S2E, Supporting Information). The substrate range assays of SNDH showed that L‐sorbosone was the most suitable substrate (Figures S2F and S3, Supporting Information).

### 2.2 Structure of the O‐SNDH

**Figure 2:**
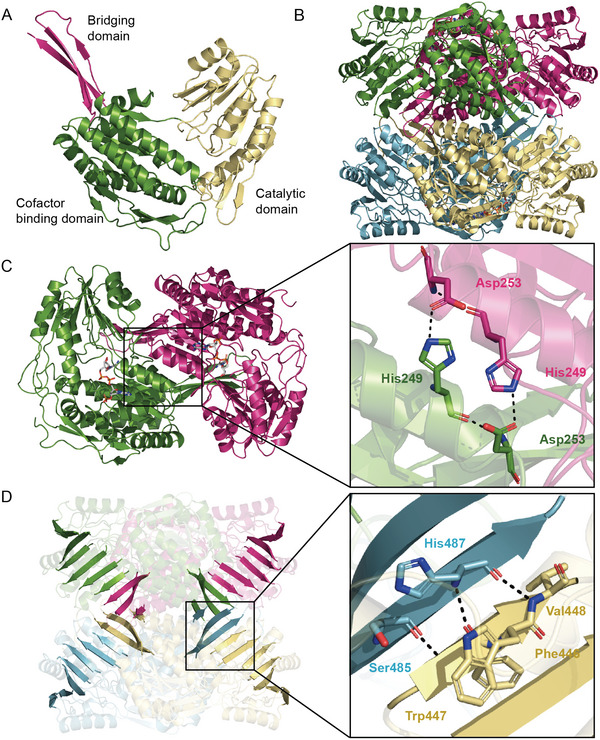
The overall structural of SNDH. A) The overall structure of oxidized SNDH (O‐SNDH). The yellow section represents the catalytic domain, the green section represents the cofactor binding domain, and the red section represents the bridging domain. B) The homotetramer of mutant SNDH C296A (C296A). C) Display the homodimer in C296A and the interaction between α‐helix in the subunits. D) Display the 10 β‐sheets of C296A and the interaction between β‐sheets in the subunits. Different colors are used to distinguish different subunits in Figure BCD. NADP^+^ is shown as a stick model in white. All stick models are colored with elements. The figures are generated by PyMOL.

**Figure 3:**
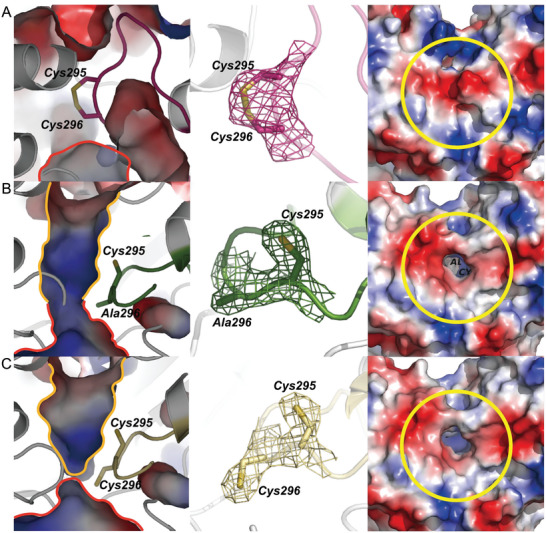
The substrate pocket characteristics of the redox state of SNDH. A) The O‐SNDH substrate pocket conformation and surface display. SNDH catalyzes the formation of a disulfide bond between Cys295 and Cys296 on the loop. At this time, the substrate pocket is closed, and the cavity of the substrate pocket is not visible in the surface display; B) The substrate pocket conformation and surface display of mutant C296A. A single Cys295 cysteine cannot form disulfide bond, and the substrate pocket is open. The substrate pocket and the cofactor pocket are connected to form a communicating cavity, and the cavity of the substrate pocket can be seen in the surface display; C) The R‐SNDH substrate pocket conformation and surface display. When DTT is added, Cys295 and Cys296 cannot form disulfide bond. The substrate is in an open conformation, and Cys296 separates the substrate pocket from the cofactor pocket. The cavity of the substrate pocket can be seen in the surface display. The substrate and cofactor pockets are circled in yellow and red, respectively. The electron density map is shown with a mesh structure. All stick models are colored with elements. The figures are generated by PyMOL.

The first structure of SNDH with the addition of NADP^+^ was identified as a monomer. Like other members of ALDH‐SF, SNDH has three typical domains: bridging domain (Red), catalytic domain (Yellow), and cofactor binding domain (Green) (**Figure** **2**A). Both the catalytic and the cofactor binding domain are composed of βαβ‐like topological structures. The bridging domain is composed of three anti‐parallel β‐sheets. There are two missing loops in SNDH (Figure S4, Supporting Information), loop A (Thr241–Asn255) in the cofactor domain and loop B (Glu457–Trp468) adjacent to the catalytic domain. Although the overall structure of SNDH is similar to other members of ALDH‐SF, a significant difference is that the substrate pocket in the monomeric structure has a “closed conformation” Because the substrate pocket was not observed in the surface display (**Figure** **3**A). This closed conformation results from the extension of the catalytic loop (Ile288–Ser300) toward the outside of the protein. A disulfide bond is formed between the catalytic cysteine (Cys296) and an adjacent cysteine (Cys295) in the catalytic loop. This interesting phenomenon suggests that the catalytic loop of SNDH has motility, which may be controlled by the redox states of Cys295 and Cys296, thus regulating the opening and closing of the substrate pocket. The closed‐state structure of the SNDH substrate pocket is referred as O‐SNDH in the following parts.

### 2.3 Structure of the Mutant C296A

To prove the hypothesis that the catalytic loop is dynamically regulated by a disulfide bond, the catalytic residue Cys296 was mutated into Ala. Mutant C296A was resolved in the form of a homotetramer, which can be described as a dimer of homodimers, and there was no obvious difference in the main chain structure between different subunits (Figure 2B,C). There are two sets of interactions between two subunits in each dimer. One consists of an α‐helix (α‐α interaction) between the two subunits, His249 in one subunit and Asp253 in the other subunit form hydrogen bonds (Figure 2C); the other group consists of β‐sheets in the bridging domain of one subunit and β‐sheets in the catalytic domain of the other subunit (β‐β interaction), forming a 10‐chain β‐sheet structure, the interaction site consists of Phe446, Trp447, and Val448 in one subunit and Ser485 and His487 in the other subunit (Figure 2D).

**Figure 4:**
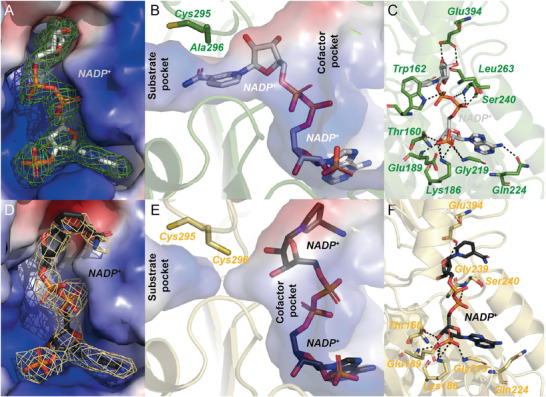
The cofactor‐binding properties to SNDH. A) The electrostatic potential surfaces of the cofactor channels of mutant C296A. NADP^+^ is shown as white stick. The electron density map is shown with a mesh with green. B) Effects of Ala296 on NADP^+^ binding position. C) Residues interacting with NADP^+^ in mutant C296A. Dotted lines denote possible hydrogen bonds. D) The electrostatic potential surfaces of the cofactor channels of reduced SNDH (R‐SNDH). NADP^+^ is shown as black stick. The electron density map is shown with a mesh with yellow. E) Effects of Ala296 on NADP^+^ binding position. F) Residues interacting with NADP^+^ in R‐SNDH. Dotted lines denote possible hydrogen bonds. All stick models are colored with elements. The figures are generated by PyMOL.

As expected, compared with O‐SNDH, the catalytic loop of the mutant C296A shows a significant difference. Since Cys295 in the catalytic loop cannot form the disulfide bond without Cys296, the catalytic loop enters the enzyme, which makes the substrate pocket present an open conformation (Figure 3B). In addition, the cofactor pocket has a wide positively charged opening on the protein surface, which can accept the adenosine monophosphate (AMP) part and pyrophosphate group of NADP^+^ (**Figure** **4**A). The cofactor pocket is connected with the substrate pocket forming a cofactor‐substrate‐channel, in which the nicotinamide ring of NADP^+^ is located (Figure 4B). In the cofactor pocket of C296A, Thr160, Lys186, and Gln224 stabilize the adenosine ribose. Lys186, Gly219, and the backbone amide groups of Glu189 interact with the 2'‐phosphate group. Trp162 and the backbone amide group of Ser240 hydrogen bonded to the pyrophosphate moiety. Leu263 and Glu294 stabilized the nicotinamide ribose by hydrogen bonding (Figure 4C).

### 2.4 Structure of the R‐SNDH

To verify whether the formation of the disulfide bond is a key factor affecting the motion of the catalytic loop, the crystal structure of R‐SNDH was analyzed by adding dithiothreitol (DTT) to the crystallization conditions. As with the C296A mutant, R‐SNDH was also resolved in a homotetrameric state, and each subunit had the same mode of action as mutant C296A (Figure S5, Supporting Information). As expected, R‐SNDH has an open substrate pocket, in which Cys295 and Cys296 cannot form the disulfide bond, the sulfhydryl of Cys296 toward to the cofactor pocket, which called ‘resting’ state, blocking the cofactor‐substrate‐channel (Figure 3C). The pose of the cofactor NADP^+^ in R‐SNDH is different from that in C296A. In R‐SNDH, the nicotinamide ring of NADP^+^ is extends to a solvent region (Figure 4D), this pose should be that the sulfhydryl of Cys296 blocks the cofactor‐substrate‐channel, causing the cofactor channel of SNDH to have no space for the nicotinamide ring of NADP^+^ (Figure 4E). Compared with C296A, the interactions with NADP^+^ in R‐SNDH is changed. In R‐SNDH, although the nicotinamide ring has a larger displacement, it still has a hydrogen bond with Glu294. The pyrophosphate part loses the interaction with Trp162, and forms a hydrogen bond with Gly239 (Figure 4F). Subsequently, the content of free Cys residues in R‐SNDH and O‐SNDH was determined. The results showed that the content of free cysteine in R‐SNDH (0.089 ± 0.003) was higher than that in O‐SNDH (0.027 ± 0.003). This indicates that O‐SNDH is capable of forming disulfide bonds, which leads to the formation of a closed conformation.

### 2.5 Dynamic Regulation and Catalytic Mechanisms of SNDH

**Figure 5:**
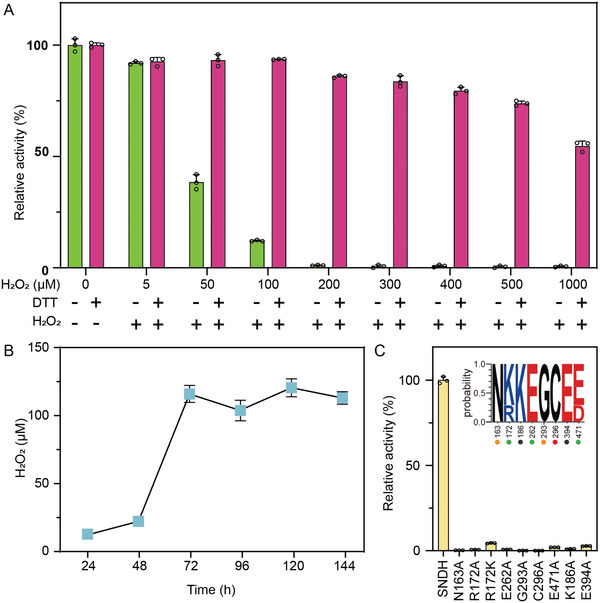
SNDH senses ROS in vitro and determines the key amino acids of catalysis. A) SNDH senses ROS in vitro. SNDH specific activity was measured in an oxidized and reduced environment by treating with H_2_O_2_ and DTT, respectively; B) Detection of intracellular H_2_O_2_ during fermentation of *G. oxydans*; C) SDNH key catalytic site mutation ratio enzyme activity detection.

**Figure 6:**
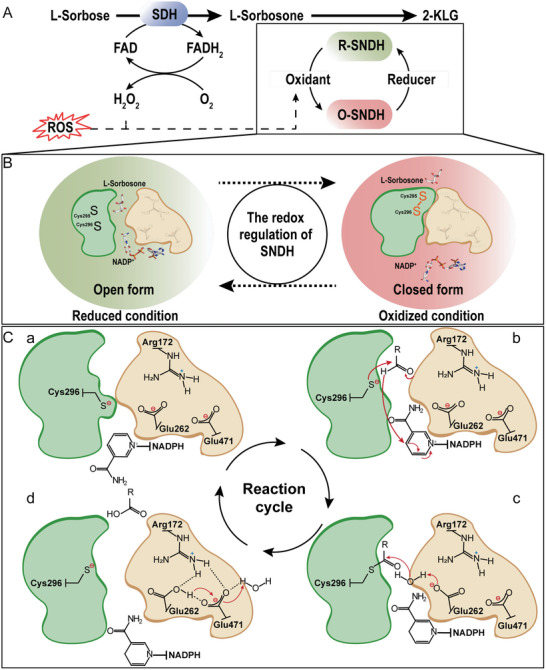
The redox regulation and the catalytic mechanism of SNDH. A) The redox regulation mechanism of SNDH. SDH: *G. oxydans* WSH‐004 L‐sorbose dehydrogenase; R‐SNDH: reduced SNDH; O‐SNDH: oxidized SNDH; Oxidant: oxidizing agent that can oxidize R‐SNDH to O‐SNDH in vivo; ROS: reactive oxygen species in vivo; Reducer: reducing agent that can reduce O‐SDNH to R‐SNDH in vivo. The solid arrow represents the reaction direction, and the dashed arrow represents the possible direction of action. B) SNDH redox switch‐mediated reaction mechanism. In an oxidizing environment, the catalytic residue Cys296 is oxidized to form a disulfide bond with Cys295, and SNDH is in the “closed conformation”. When switched to a reducing environment, the disulfide bond between Cys295 and Cys296 is broken, and SNDH adopts an “open form”, which can perform catalytic effects. C) The catalytic mechanism of SNDH is proposed. a ‘resting’ state. The cofactor channel and the substrate channel are disconnected by the blocking of the catalytic residue Cys296. b ‘Hydride transfer’ state. When the substrate and the cofactor enter their respective pockets, the nucleophilic Cys296 attacks the substrate to form a four‐sided intermediate, and at the same time transfers the protons on the substrate to NADP^+^. c **‘**Hydrolysis’ state. Glu262 captures a proton from the water molecule and transmits it to the solution through the electronic relay system (Glu262‐Arg172‐Glu471). The OH^−^ ions combine with the tetrahedron to complete the substrate catalysis. d ‘Release’ state. NADPH and the product are released, and the enzyme molecule returns to a resting state. Green represents the catalytic domain, and yellow represents the cofactor domain. The red arrow shows the direction of electron transfer. The opening in the middle of the domain represents the cofactor channel and the substrate channel.

Many studies have shown that the reactive oxygen species (ROS) produced in vivo can regulate redox‐mediated proteins.^[^ ^27^, ^28^, ^29^, ^30^ ^]^ To verify whether SNDH could be regulated by the redox environment, the sensitivity of SNDH to different concentrations of H_2_O_2_ was tested. The results showed that SNDH almost loses its activity when H_2_O_2_ reaches 200 µm (**Figure** **5**A). After adding 3 mm DTT, the activity was significantly restored. In addition, the intracellular H_2_O_2_ content of *G. oxydans* was remained above 100 µm after 72 h (Figure 5B), which may inactive the SNDH. Based on the structural information and its role in the biosynthesis of 2‐KLG, a regulation mechanism of SNDH by an in vivo redox status was proposed (**Figure** **6**A). In *G. oxydans* WSH‐004, L‐sorbose dehydrogenase oxidizes L‐sorbose into L‐sorbosone and generates FADH_2_, using oxygen as the final electron acceptor to reduce FADH_2_ and generate H_2_O_2_. In addition, a large number of ROS are generated in numerous oxidation‐reduction reactions.^[^ ^31^, ^32^ ^]^ The presence of ROS can make Cys295 and Cys296 on the catalytic loop form a disulfide bond, which make R‐SNDH turns into O‐SNDH without catalytic activity. When the O‐SNDH is in a suitable reducing environment, it can be transformed into R‐SNDH with catalytic activity (Figure 6B).

Furthermore, the molecular catalytic mechanism of SNDH through mutation experiments was verified. Multiple sequence alignments showed that the potential active site residues (Cys296, Glu262, Asn163, and Gly293) in SNDH are highly conserved in ALDH‐SF, and differences only occur at Arg172 and Glu471 (Figure 1D). When these amino acid residues were mutated to Ala, the enzyme activity of the mutant was extremely low or even disappeared (Figure 5C). Subsequently, the catalytic mechanism (Figure 6C) of SNDH was proposed by the structural analysis and the reports on the catalytic mechanism of ALDH.^[^ ^20^, ^21^, ^33^ ^]^ In the structure of R‐SNDH, the nicotinamide ring of NADP^+^ cannot enter the cofactor pocket because the catalytic residue Cys296 occupies a part of the cofactor pocket, and Cys296 should be in a ‘resting’ state.^[^ ^20^, ^21^ ^]^ In the mutant C296A, since Ala296 has no sulfhydryl groups, an enough space can be left for NADP^+^ to enter the substrate‐cofactor‐channel. This indicates that SNDH may first accept the substrate during the reaction, which makes the sulfhydryl group of Cys296 point to the substrate pocket. Due to the deflection of the sulfhydryl of Cys296, the nicotinamide ring of NADP^+^ can correctly enter the cofactor channel to complete the electron transfer. Subsequently, Glu262 acts as a general base, activating water molecules to attack the thioester intermediate, complete the substrate reaction catalysis, and release the corresponding carboxylic acid product and NADPH. Another proton is transferred to Glu262, and then through the proton transfer system composed of Arg172 and Glu471, Glu262 is deprotonated and the proton is eventually transferred to the solution.

### 2.6 Exploring the Dynamic Regulatory Mechanism of SNDH

To investigate the “open to closed” transformation of O‐SNDH and the “closed to open” transformation of R‐SNDH, steered molecular dynamics simulations and umbrella sampling were performed. The calculation results show that the closing of the catalytic loop in O‐SNDH require a free energy of 43.4 kcal mol^−1^ (20 to 5 Å in reaction coordinate) (Figure S6A,B, Supporting Information). For the opening of the catalytic loop in R‐SNDH, the opening process require a free energy of 62.9 kcal mol^−1^ (5 to 20 Å in reaction coordinate) (Figure S6C,D, Supporting Information), indicating the opening process is less favorable than the closing process. The difference in the value of free energy of closing and opening process could be cause by the interaction between the catalytic loop and the nearby α‐helix (Lys96–Ala110), and the interaction between the catalytic loop and the newly rebuilt loop (Glu457–Trp468) in O‐SNDH.

**Figure 7:**
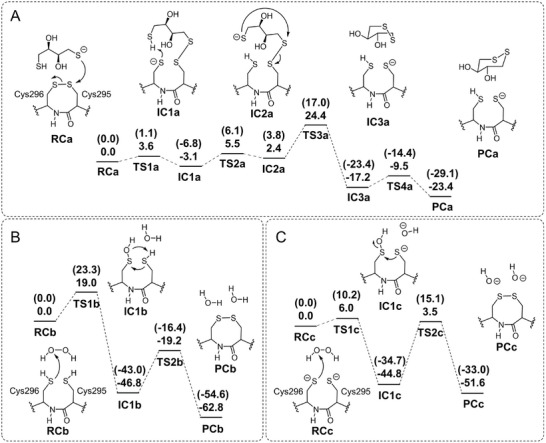
The cleavage and formation processes of disulfide bond between Cys295 and Cys296. A) The cleavage processes of disulfide bond mediated by DTT. B) The formation processes of disulfide bond between protonated Cys295 and Cys296 mediated by H_2_O_2_. C) The formation processes of disulfide bond between deprotonated Cys295 and Cys296 mediated by H_2_O_2_. The relative energies are given as the sum of single‐point energies, dispersion corrections, solvation effects and ZPE corrections. The relative energies calculated at B3LYP/def2‐SVP level are shown in parentheses. All energies are given in kcal mol^−1^.

In order to investigate the cleavage and formation of disulfide bond between Cys295 and Cys296, the quantum chemical calculations were performed based on the previous studies.^[^ ^34^, ^35^, ^36^, ^37^ ^]^ In the cleavage processes of disulfide bond mediated by DTT (**Figure** **7**A; Figure S7, Supporting Information), first, the sulfur anion of DTT attacks the sulfur atom of Cys295 in reactant complex (RCa), which has an energy barrier of 3.6 kcal mol^−1^ from transition state (TS1a) to RCa and leads the formation of intermediate complex (IC1a). After the proton transfer process from IC1a to IC2a, hexatomic ring was formed in IC3a with an energy barrier of 22.0 kcal mol^−1^ (IC2a to TS3a). Last, after a conformational change of the hexatomic ring, the final product complex (PCa) is generated with an energy of −23.4 kcal mol^−1^ relative to RCa. The overall energy barrier for the cleavage processes is 27.5 kcal mol^−1^ (IC1a to TS3a).

In the formation processes of disulfide bond mediated by H_2_O_2_ (Figure 7B; Figure S8A, Supporting Information), the sulfur atom on the sulfhydryl group of Cys296 attacks the oxygen atom in H_2_O_2_, which has an energy barrier of 19.0 kcal mol^−1^ from reactant complex (RCb) to transition state (TS1b). Then, the intermediate complex (IC1b) is generated with an energy of −46.8 kcal mol^−1^ relative to RCb. Last, after the disulfide bond is formed, the product complex (PCb) is generated with an energy of −62.8 kcal mol^−1^ relative to RCb. The rate‐determine step is the disulfide bond formation step, which has an energy barrier of 27.6 kcal mol^−1^ (IC1b to TS2b). As the sulfhydryl group of cysteine could be deprotonated at the experimental pH (pH = 8.0), the disulfide bond formation processes between protonated Cys295 and Cys296 were also investigated (Figure 7C; Figure S8B, Supporting Information). The calculation results show the rate‐determine step is also the disulfide bond formation process, which has an energy barrier of 48.3 kcal mol^−1^. The high energy barrier could be attributed to the generation of second hydroxide ion in the second transition state or the unfavorable interactions between the two deprotonated cysteines and hydroxyl group in the second transition state. In short, the overall energy barrier of disulfide bond cleavage processes (27.5 kcal mol^−1^) is similar to that of disulfide bond formation processes (protonated cysteines, 27.6 kcal mol^−1^), indicating the disulfide bond is reversible.

### 2.7 Steric Hindrance Modification to Improve the Catalytic Activity of SNDH

**Figure 8:**
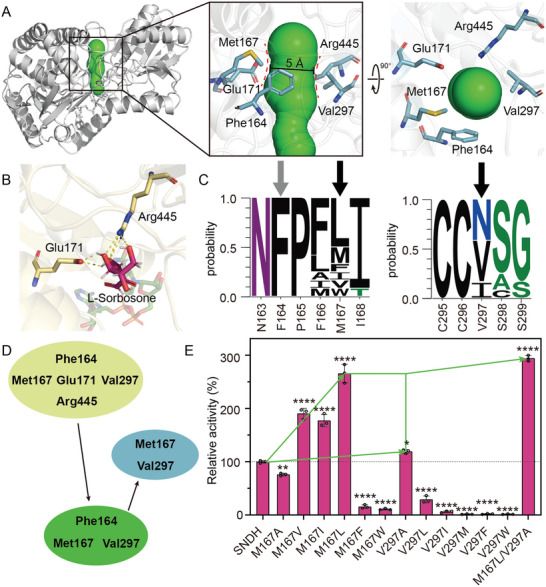
Identifying the mutation site of SNDH and its engineering. A) Display of SNDH substrate tunnel and the residues of its bottleneck. B) The display of hydrogen bond between L‐sorbosone and the residues of SNDH substrate tunnel. C) Sequence conservation analysis of residues at SNDH substrate tunnel bottlenecks. D) Screening of key mutagenesis sites. E) Determination of SNDH and its mutant activity.

Based on the structure, modification of the steric hindrance of SNDH to increase catalytic activity has been attempted. First, the substrate channels of SNDH were analyzed with CAVER 3.0.3. The channel shows an obvious bottleneck area composed of Phe164, Met167, Glu171, Val297, and Arg445 (**Figure** **8**A). In order to expand the bottleneck area, CDOCKER was first used to dock L‐sorbosone in the substrate channel of SNDH. Since Glu171 and Arg445 have hydrogen bond with L‐sorbosone, they were excluded (Figure 8B). Conservative sequence analysis of SNDH and ALDH‐SNDH family members showed that Phe164 was highly conservative in ALDH‐SNDH family (Figure 8C). Besides, Met167 and Val297 were selected as mutation sites to release the bottleneck region of SNDH substrate tunnel (Figure 8D).

The following principles were used for the construction of SNDH mutants. 1) Met167 and Val297 were replaced by bulky residues to investigate the effect of channel narrowing at the SNDH bottleneck area on enzyme activity. Therefore, the mutant M167F, M167W, V297L, V297I, V297M, V297F, and V297W was constructed. 2) Met167 and Val297 were replaced by small residues to investigate the effect of SNDH bottleneck region release on enzyme activity. Therefore, mutants M167A, M167V, M167I, M167L, and V297A were constructed. Subsequently, the specific activity of the mutant was tested. Excitingly, we observed the expected results in the mutants. Specifically, the activity of mutants constructed according to principle 1) decreased to varying degrees; while the mutants constructed according to principle 2), except M167A, showed increased activity (Figure 8E). Among them, the specific activity of M167L was increased by 2.7‐fold, and the specific activity of V297A was increased by 1.2‐fold. Finally, M167L/V297A was constructed, which the specific activity increased 2.9‐fold than SNDH (Figure 8E).

Subsequently, the kinetic parameters of SNDH and representative mutants (M167L, V297A, and M167L/V297A) were determined to investigate the effect of the mutants on the catalytic efficiency of L‐sorbosone. The results showed, compared with SNDH, the *k*_cat_/*K*_m_ of mutants M167L, V297A, and M167L/V297A were increased by 1.82, 1.31, and 2.33‐fold, respectively. Among them, the increase of M167L activity is mainly due to the increase of *k*_cat_ value (1.66‐fold higher than SNDH), and the increase of V296A activity is mainly due to the decrease of *K*_m_ value (1.21‐fold lower than SNDH). The increase in activity of the combined mutant M167L/V297A was attributed to a 1.38‐fold lower in *K*_m_ value and a 1.68‐fold increase in *k*_cat_ value than SNDH (**Table** **1**). These results indicate that the increase of specific activity of M167L, V297A, and M167L/V297A was attributed to the decrease of *K*_m_ value and the increase of *k*_cat_ value, respectively.

**Table 1:** Kinetic parameters of SNDH and its mutants

Enzyme	*K*_m_ [mm]	*k*_cat_ [s^−1^]	*k*_cat_/*K*_m_ [mm ^−1^ s^−1^]
SNDH	1.056	1.795	1.700
M167L	0.9629	2.973	3.088
V296A	0.8750	1.955	2.234
M167L/V297A	0.7635	3.024	3.961

## 3 Discussion

At present, the research on the one‐step fermentation for vitamin C production is focused mainly on the metabolic engineering of the 2‐KLG synthesis pathway, and few studies have understood the catalytic mechanism from the structure of the key dehydrogenases involved.^[^ ^2^, ^38^, ^39^ ^]^ This study showed that SNDH not only has stronger substrate specificity for L‐sorbosone, and requires the cofactor NAD(P)^+^ is more widespread than PQQ of the classical two‐step fermentation route, making it more suitable as an L‐sorbosone specific catalytic enzyme for metabolic engineering. By analyzing the crystal structure, the redox regulation mechanism of SNDH mediated by reversible disulfide bond was discovered, and the molecular catalytic mechanism of SNDH was also proposed. Moreover, based on the analysis of the SNDH substrate channel, the mutant with enhanced activity was obtained. This work contributes to understanding the physiological control mechanism of SNDH in *G. oxydans* and enriches the catalytic mechanism of ALDH‐SF, provides a theoretical basis for the application of SNDH or ALDH in the future.

The members of ALDH‐SF have similar structural backbones, conserved catalytic residues and similar physicochemical properties.^[^ ^17^, ^40^ ^]^ Some of the previous studies have shown that the quaternary structure is necessary for ALDHs. Mutations related cofactor binding defects could lead to dissociation of the tetrameric structure, could thus reduce or even inactivate the enzyme activity.^[^ ^22^, ^41^ ^]^ The hydrogen bond between subunits is the key factor to form a tetramer.^[^ ^42^, ^43^ ^]^ However, O‐SNDH does not have any residue mutation and does not involve hydrogen bond loss. In principle, it should not be solved in the monomer form. Therefore, the electron density diagram of O‐SNDH was observed, and it was found that O‐SNDH also existed with tetramer as the basic unit (Figure S9A,B, Supporting Information). In addition, the dynamic light scattering shows that the particle size of O‐SNDH and R‐SNDH in solution were similar, both in the range of 11–12 nm (Figure S9C, Supporting Information). This indicates that O‐SNDH folds as a tetramer in solution, but it is more suitable for analysis in the form of monomer structure. It also indicates that the inactivate of O‐SNDH is due to the closure of the substrate pocket, rather than the dissociation of the tetramer structure.

The catalytic residue Cys in the ALDH family is absolutely conserved.^[^ ^44^ ^]^ In the resolved structures, the Cys is found to have two different conformations. When the sulfhydryl of Cys296 points to the cofactor pocket, it is called the ‘resting’ state; when the sulfhydryl of Cys296 points to the substrate pocket, it is called the ‘attacking’ state.^[^ ^20^ ^]^ In addition, when the nicotinamide ring of NAD(P)^+^ is located in the cofactor‐substrate‐channel, it is called the ‘hydride‐transfer’ pose, and when the nicotinamide ring of NAD(P)^+^ is extends to a solvent region, it is called the ‘hydrolysis’ pose.^[^ ^45^ ^]^ Our study showed, the Cys296 is in the ‘resting’ state in O‐SNDH. In addition, it was also found that the state of Cys296 can regulate the connection and interruption of cofactor channels and substrate channels, which in turn affects the binding posture of NADP^+^. Some metal ions can promote the activity of ALDHs.^[^ ^46^ ^]^ Among them, Mg^2+^ is considered to regulate the dissociation of cofactors by combining with the pyrophosphate group of NAD(P)^+^, thereby affecting the activity of ALDHs.^[^ ^47^ ^]^ Mg^2+^ may also regulate SNDH activity in the same way.

Dynamic regulatory mechanisms in the ALDH‐SF are not often discovered. Some studies have found that ROS can reversibly inactivate ALDH, but there is no structural data to prove it.^[^ ^48^, ^49^ ^]^ By carefully reviewing the structure of ALDHs in the PDB database, it is found that most of the solved structures have an open substrate pocket.^[^ ^20^, ^23^, ^26^ ^]^ Only succinic semialdehyde dehydrogenase (SSADH, PDB code: 2W8N) from *Homo sapiens* has a closed substrate pocket conformation (Figure S10, Supporting Information), and has a similar dynamic regulation mechanism based on the redox properties of Cys.^[^ ^50^ ^]^ The main difference between SNDH and SSADH is the position of the non‐catalytic Cys. In SSADH, the non‐catalytic Cys342 does not participate in the composition of the substrate pocket (Figure S11A, Supporting Information). The mutation to Ala can prevent SSADH from forming an oxidation state in which the substrate pocket is closed, thereby enhancing the activity. In SNDH, the non‐catalytic Cys295 is part of the substrate pocket of SNDH (Figure S11B, Supporting Information), and the mutation of Cys295 will reduce the activity of SNDH for L‐sorbosone (Figure S11C, Supporting Information). Multiple sequence alignment of SNDH subfamily members showed that non‐catalytic Cys residues were conserved in most members, only the SNDH from *Paracoccus denitricans* is replaced by Glu (Glu284). However, the SNDH from *P. denitricans* has an additional Cys (Cys287) at the adjacent position (Figure S12, Supporting Information). This may indicate that the non‐catalytic Cys near the catalytic Cys plays an important role in SNDH subfamily members.

The study of the catalytic mechanism assists in understanding how enzymes function. In the ALDH family, many structures have been analyzed and the catalytic mechanism has been studied. Gonzalez et al. studied the structure of betaine aldehyde dehydrogenase (BADH) and proposed a mechanism for a proton relay system in BADH.^[^ ^21^ ^]^ By studying NAD(P)^+^‐dependent dehydrogenase from *Vibrio variabilis* JCM 19239, Wang et al. proposed a catalytic mechanism to regulate the connection and interruption of the cofactor channel and substrate channel through the conformational changes of Cys282 and Glu248.^[^ ^20^ ^]^ The residues of SNDH electronic relay system have conserved chemical properties with the members of the ALDH family, but the types are different. For example, Lys172 in SNDH corresponds to Arg162 in BADH from *Pseudomonas aeruginosa*;^[^ ^21^ ^]^ Glu471 in SNDH corresponds to Asp883 in aldehyde dehydrogenase from *Rattus norvegicus*.^[^ ^51^ ^]^ This study shows that SNDH from *G. oxydans* WSH‐004 also has a similar catalytic mechanism, even though the catalytic residues are not fully conserved. Furthermore, the high pH dependence of optimal activity also supports the mechanism of proton transfer into solution.

Based on structure resolution, changing the steric hindrance to reshape the pocket is an effective method to enhance enzyme activity. For example, enlarging the substrate entry and binding pockets.^[^ ^52^, ^53^ ^]^ Moreover, the construction of small intelligent libraries through rational selection for combinatorial mutation, the efficiency of mutant screening can be improved, such as triple‐code saturation mutation,^[^ ^54^, ^55^, ^56^ ^]^ multi‐library combination^[^ ^53^ ^]^ and iterative saturation mutagenesis,^[^ ^57^ ^]^ etc. For ALDHs, by mutating non‐catalytically required Cys, the redox regulation of ALDH can be broken, improving the catalytic activity ^[^ ^50^ ^]^. However, this method is not suitable for engineering SNDH (Figure S11C, Supporting Information). This study shows that using CAVER to analyze SNDH is appropriate. Subsequently, through molecular docking and amino acid conservation analysis, Met167 and Val297 were finally identified as mutation residues, and a small mutation library was constructed, which improved the screening efficiency.

The members of ALDH‐SF play a very important role in the metabolism of aldehydes, which can catalyze the formation of carboxylic acids from corresponding aldehydes.^[^ ^40^, ^58^ ^]^ Previous studies have elucidated the crystal structures, catalytic mechanisms and regulatory mechanisms of some members of ALDH‐SF.^[^ ^24^, ^59^, ^60^ ^]^ This study is the first time to analyze the structure of members of the SNDH subfamily in ALDH‐SF. The results show that SNDH has two forms, one is oxidized form that closes the substrate pocket and other is reduced form that opens the substrate pocket. Both forms are mediated by reversible disulfide bond and regulated by the redox environment. Combined with the vitro experiments and previous reports, the dynamic regulation and catalytic mechanisms of SNDH were proposed, respectively. Finally, the activity of SNDH was enhanced by engineering the substrate tunnel. These results could provide a theoretical basis for understanding the molecular mechanism of *G. oxydans* SNDH in one‐step fermentation to produce 2‐KLG, and the rational design and application of ALDH family enzymes in the future.

## 4 Experimental Section

### Expression and Purification of SNDH in *E. coli* BL21 (DE3)

The gene sequence of SNDH was PCR‐amplified from the genomic DNA of *G. oxydans* WSH‐004 and inserted into pET‐28a(+), resulting in pET‐SNDH. The primers used are listed in Table S2 (Supporting Information). The purified plasmids were transformed into *E. coli* BL21(DE3) and cultivated in LB fermentation medium at 37 °C to an OD_600_ of 0.6–0.8. The culture temperature was lowered to 16 °C and isopropyl β‐D‐thiogalactoside (IPTG) was added to a final concentration of 0.5 mm. The culture was continued for 18 h to induce protein expression. The cultured cells were collected and resuspended in buffer A (25 mm Tris, 300 mm NaCl, 25 mm imidazole, pH = 8.0). A homogenizer was used for cell lysis. The lysis was centrifuged at 12,000 *g* for 2 h. The supernatant was passed through a Ni‐NTA column and equilibrated with buffer A. Buffer B (25 mm Tris, 300 mm NaCl, 500 mm imidazole, pH = 8.0) was used to elute and collect the target protein. The target protein was concentrated and then purified by using a Superdex 200 pg 26/600 column equilibrated with buffer C (25 mm Tris, 150 mm NaCl, pH = 8.0). SDS‐PAGE was used to assess protein purity. The protein concentration was determined by the BCA method.

### Mutagenesis Experiments

All mutants were conducted by whole plasmid PCR using 2 × Phanta Max Master Mix (Vazyme Biotech Co., Ltd). The primers used are listed in Table S3 (Supporting Information). After *Dpn*I digestion at 37 °C for 30 min, the PCR products were transformed into competent *E. coli* BL21 (DE3) cells (Sangon Biotech (Shanghai) Co., Ltd.) and plated into LB agar plates supplemented with 50 µg mL^−1^ kanamycin. Mutants were verified with Sanger sequencing, mutant expression and purification methods are the same as for SNDH.

### Crystallization, Data Collection, and Structure Determination

The purified SNDH and C296A mutant were concentrated to 15–20 mg mL^−1^. The sitting drop vapor diffusion technique was used to screen the crystals using crystallization kit (Hampton Research Protein Crystallization Kit). The crystals of O‐SNDH appeared in 0.1 m BIS‐TRIS pH 5.5 and 1 mM NADP^+^. The crystals of R‐SNDH appeared in 2.8 m sodium acetate trihydrate pH 7.0, 1 mm NADP^+^ and 1 mm DTT. The crystals of mutant C296A appeared in 1.0 m succinic acid pH 7.0; 0.1 m HEPES pH 7.0; 1% PEGME 2000 and 1 mm NADP^+^. The crystals were transferred to a pool containing 30% glycerin and quickly cryoprotected with liquid nitrogen. The data were collected at the BL17U and BL19U1 beamlines of the Shanghai Synchrotron Radiation Facility. HKL2000/HKL3000^[^ ^61^ ^]^ was used for data processing. The NAD^+^ dependent ALDH from *Sinorhizobium meliloti* was used as the template (PDB code: 3U4J). All crystal structures were determined by the molecular replacement method in CCP4.^[^ ^62^ ^]^ WinCoot^[^ ^63^ ^]^ and PHENIX^[^ ^64^, ^65^ ^]^ were used to refine the structure. Table S4 (Supporting Information) lists the data information related to the structure. The atomic coordinates of O‐SNDH, R‐SNDH and mutant C296A were stored in the PDB. The crystallographic information was placed in the Protein Data Bank (PDB) with ID numbers 7W5L, 7W5N, and 7W5K.

### Biochemical Characterization, Activity and Kinetic Parameters Analysis In Vitro

The enzymatic activity of SNDH was calculated by detecting the increase of the absorption peak of NAD(P)H at 340 nm by using a BioTek microplate spectrophotometer with 96‐well plates. The total reaction system included target enzyme, 2 µl; L‐sorbosone (3 mm); NAD(P)^+^ (2 mm); and made up to 200 µl with buffer C. One unit of enzyme activity was defined as the amount of enzyme that forms 1 µmol of NADPH per minute at 30 °C, which was equivalent to the conversion of 1 µmol substrate per minute. Specific enzyme activity was the ratio of enzyme activity to protein concentration.

Enzyme kinetic assays of SNDH with the concentrations of NAD^+^ and NADP^+^ from 0 to 8 mm and a fixed concentration of 3 mm for L‐sorbosone. Enzyme kinetic assays of SNDH using L‐sorbosone at concentrations from 0 to 5 mm and NAD^+^ and NADP^+^ at a fixed concentration of 2 mm. Kinetic parameters were calculated by nonlinear regression to fit directly to the Michaelis‐menten equation using the GraphPad Prism 9.0.0 (GraphPad Software, La Jolla, CA, USA).

The subsequent experiments are all expressed by the relative specific enzyme activity. The substrate spectrum of SNDH was assayed by using different substrates (glyoxal, L‐sorbitol, phenylacetaldehyde, hexanal, 3‐methyl‐2‐butenal, cinnamaldehyde, L‐octanal, nonanal, benzaldehyde, N‐heptanal, acetaldehyde, leaf‐aldehyde, L‐sorbose, L‐rhamnose, glycerol, N‐hexane, N‐propanol, maltose, methanol, isopropanol, L‐ascorbic acid, D‐Fructose, gluconolactone, D‐glucose, L‐arabinitol, sucrose, D‐sorbitol, 2‐KLG, D‐xylose, mannitol, L‐malic acid, inositol D‐xylitol, ethylene glycol and ethanol). The highest specific enzyme activity was set to 100% to calculate the relative specific enzyme activity.

The different pH buffers (acetic acid‐sodium acetate buffer (20 mm): pH 3.0–6.0 (increments of 0.5 °C); potassium dihydrogen phosphate‐dipotassium hydrogen phosphate buffer (20 mm): pH 6.0–8.0 (increments of 0.5 °C); glycine‐sodium hydroxide solution (20 mm): pH 8.0–12.0 (increments of 0.5 °C) were used to determine the optimal reaction pH of SNDH.

The optimal reaction temperature of the enzyme was measured in the range of 20–65 °C (increments of 5 °C). The thermal stability was tested by incubating the enzyme for 10 min in the range of 25–70 °C (increments of 5 °C).

Metal ion determination uses buffer C without NaCl as the control buffer. The effect of metal ions on the catalytic activity of SNDH was studied by adding different concentrations (0.1 and 1 mm) and different types of metal ion (ZnCl_2_, MgCl_2_, CdCl_2_, FeSO_4_, CaCl_2_, MnSO_4_, CoCl_2_, NiCl_2_, CuCl_2_, CrCl_3_, Fe_2_(SO4)_3_, NaCl and KCl) to the system.

To investigate how SNDH senses ROS, the purified SNDH was treated with different concentrations of H_2_O_2_ for 1 h, and then added to the reaction system for analysis. To switch the environment to a reduced state, 3 mm DTT was added to the sample after H_2_O_2_ treatment and incubated for 10 min.

O‐SNDH was prepared by adding 50 µm H_2_O_2_ to SNDH. The intracellular cysteine content was detected by a cysteine content assay kit (Solarbio), and the dynamic light scattering was used to determine the size of protein particles in solution.

Two milliliter (OD_600_≈2) of the culture broth of *G. oxydans* WSH‐004 was collected by centrifugation at 4 °C at 20,000 *g* for 10 min. The precipitation was collected, responded with 200 µL PBS/Tris‐HCl and 100 µL glass beads (diameter≈0.1 mm). The suspended cells were then lysed with FastPrep for three cycles (30 s each cycle). The lysates were then centrifuged at 4 °C at 20,000 *g* for 10 min. For detection of the H_2_O_2_ concentration, 20 µL of the supernatant was sampled and added with ADHP (10‐acetyl‐3,7‐dihydroxyphenoxazine) and horseradish peroxidase to a final concentration of 0.5 mm and 3 U, respectively, in 50 mm Tris‐HCl (pH 7.4) buffer. The fluorescence intensity was measured in the reaction with an excitation wavelength of 535 nm and an emission wavelength of 595 nm at 30 °C.^[^ ^66^ ^]^

### Tunnel Analysis

Using C296A (Chain A) as the model, analyze the tunnel of SNDH by using the default program through the CAVER 3.0.3^[^ ^67^ ^]^ plug‐in in PyMOL.^[^ ^68^ ^]^ The geometric center of the active site in SNDH wass the starting point of the tunnel.

### Molecular Docking

The structure of L‐sorbosone was obtained from PubChem (https://pubchem.ncbi.nlm.nih.gov/).^[^ ^69^ ^]^ Prepare Ligands in Discover Studio 2019 was used to prepare L‐sorbosone. CDOCKER^[^ ^70^ ^]^ was used to dock L‐sorbosone with SNDH. Top Hits was set to 100, and other values are default. The model with a reasonable docking pose and a high ‐CDOCKER_ENERGY score was finally selected for the next analysis.

### Steered Molecular Dynamics Simulations and Umbrella Sampling

As the loops Thr241–Asn255 and Glu457–Trp468 were missing in the O‐SNDH crystal (PDB ID: 7W5L), the Modeller software^[^ ^71^ ^]^ was employed to repair the missing loops based on the crystal structures of SNDH (PDB ID: 7W5L, 7W5N, 7W5K). The GPU‐accelerated *pmemd* module of Amber20^[^ ^72^ ^]^ was used for all molecular dynamics (MD) simulations of SNDH. All water molecules in SNDH crystal were retained in MD Simulations. The p*Ka* of titratable residues in SNDH were determined by using PDB2PQR^[^ ^73^ ^]^ web server (https://server.poissonboltzmann.org/pdb2pqr), and the added hydrogen atoms were visually inspected by using VMD.^[^ ^74^ ^]^ FF14SB^[^ ^75^ ^]^ and GAFF^[^ ^76^ ^]^ force filed were applied for protein residues and NADP^+^ ligand, respectively. The RESP charge was used for NADP^+^ ligand, which was calculated at HF/6‐31G(d) theory level. Then, a TIP3P^[^ ^77^ ^]^ cubic water box was used for SNDH‐NADP^+^ complex, which has a least 12 Å from the surface of protein complex to the boundary of water box. Sodium ions were added to neutralize the entire system.

For the steered MD simulations, five steps were conducted: 1) minimizing the solvents (2500 steps using the steepest descent algorithm and 2500 steps using the gradient conjugation algorithm with a restraint of 50 kcal mol^−1^ Å^−2^ for SNDH‐NADP^+^ complex); 2) minimizing the entire system (same as step 1 but without the restraint); 3) heating from 0 to 300 K in 300 ps with a restraint of 15 kcal mol^−1^ Å^−2^ using NVT ensemble; 4) equilibration at 300 K for 5 ns using NPT ensemble; 5) steered MD simulations with a pulling speed of 0.2 Å ns^−1^ between the Cα atom of C295 and E394 with a harmonic potential of 20 kcal mol^−1^ Å^−2^.

For the umbrella sampling,^[^ ^78^ ^]^ the initial configurations were extracted from the steered MD simulations, which varies from 25 to 5 Å for O‐SNDH‐NADP^+^ and 4 to 20 Å for R‐SNDH‐NADP^+^ with an interval of 0.25 Å for adjacent windows, respectively. The reaction coordinate was the same as in steered MD simulations. Each window was simulated for 5 ns with a harmonic potential of 10 kcal mol^−1^ Å^−2^ along the path of “open to closed” (for O‐SNDH‐NADP^+^) and “closed to open” (for R‐SNDH‐NADP^+^) transformation. The potential of mean force was calculated using the weighted histogram analysis method.^[^ ^79^ ^]^

### Quantum Chemical Calculations

To investigate the cleavage and formation of disulfide bond between Cys295 and Cys296, quantum chemical calculations were performed using Gaussian 16 software.^[^ ^80^ ^]^ First, the DTT and H_2_O_2_ were docked into the SNDH. A short MD simulation (5–10 ns) was then performed to relax the unfavorable steric interactions. After clustering the trajectory of MD simulations, the representative snapshot was used for model building. 5 and 13 water molecules were retained based on the interactions from DTT to protein residues and H_2_O_2_ to protein residues, respectively. Bonds from Gln294 to Cys295 and Cys296 to Val287 were truncated at the site of C─C bond. Hydrogen atoms were added to neutralize the truncated carbons. The Cα of Cys295 and Cys296 and truncated sites were kept fixed to mimic their position in reaction processes. B3LYP‐D3(BJ) density functional theory^[^ ^81^, ^82^, ^83^, ^84^ ^]^ was employed. The basis set def2‐SVP^[^ ^85^ ^]^ was used in geometry optimizations, which was denoted as BS1. To get the zero‐point energy corrections, frequency calculations were performed at the same level of geometry optimizations. To get the solvation effect of surrounding environment of enzyme, SMD solvation model^[^ ^86^ ^]^ was applied with a dielectric constant of ε = 4. The basis set def2‐TZVP^[^ ^85^ ^]^ was used to get more accurate energies in single‐point energy calculations, which was denoted as BS2. Finally, the reported energies included single‐point energies, dispersion corrections, solvation effects, and ZPE corrections.

### Statistical Analysis

All experiments in this study consist of 3 replicates. GraphPad Prism 9.0.0 was used for statistical analysis. The statistical evaluations used one‐way ANOVA, followed by Dunnett tests. The results were considered statistically significant at *P < 0.05.

## Conflict of Interest

The authors declare no conflict of interest.

## Author Contributions

D.L. and Z.D. contributed equally to this work. D.L., Z.D., X.H., Y.C., and D.Y. performed experiments and data analysis. D.L., X.W., Z.Q., Y.R., and J.Z. wrote the manuscript and conceived the study. J.C., and J.Z. coordinated the project.

## Supporting information

Supporting Information

Supporting Information

## Data Availability

Research data are not shared.
