# Supplementary material for: Structural Insight into the Catalytic Mechanisms of an L‐Sorbosone Dehydrogenase
Source: Adv Sci (Weinh). 2023 Sep 7;10(30):2301955. doi: 10.1002/advs.202301955 (PMC10602560; doi:10.1002/advs.202301955)
Supplement: Supplementary file 1 — Supporting Information [file ADVS-10-2301955-s002.pdf]

ADVANCED SCIENCE
Open Access

Supporting Information

for *Adv. Sci.*, DOI 10.1002/advs.202301955

Structural Insight into the Catalytic Mechanisms of an L-Sorbosone Dehydrogenase

*Dong Li, Zhiwei Deng, Xiaodong Hou, Zhijie Qin, Xinglong Wang, Dejing Yin, Yue Chen, Yijian Rao, Jian Chen and Jingwen Zhou**

Supplementary Information

Structural insight into the catalytic mechanisms of an L-sorbosone dehydrogenase

Dong Li[1,2,3,5], Zhiwei Deng[3,5], Xiaodong Hou[3], Zhijie Qin[1,2,3], Xinglong Wang[1,2,3], Dejing Yin[3], Yue Chen[3], Yijian Rao[3], Jian Chen[1,2,3,4], Jingwen Zhou[1,2,3,4*]

[1] Engineering Research Center of Ministry of Education on Food Synthetic Biotechnology, Jiangnan University, 1800 Lihu Road, Wuxi, Jiangsu 214122, China;

[2] Science Center for Future Foods, Jiangnan University, 1800 Lihu Road, Wuxi, Jiangsu 214122, China;

[3] Key Laboratory of Industrial Biotechnology, Ministry of Education and School of Biotechnology, Jiangnan University, 1800 Lihu Road, Wuxi, Jiangsu 214122, China;

[4] Jiangsu Province Engineering Research Center of Food Synthetic Biotechnology, Jiangnan University, Wuxi 214122, China.

[5] These authors contributed equally: Dong Li, Zhiwei Deng.

[*] Corresponding author: Jingwen Zhou

Mailing address: Science Center for Future Foods, Jiangnan University, 1800 Lihu Road, Wuxi, Jiangsu 214122, China

Phone: +86-510-85914371, Fax: +86-510-85914371

E-mail: zhoujw1982@jiangnan.edu.cn

Supplementary Figures

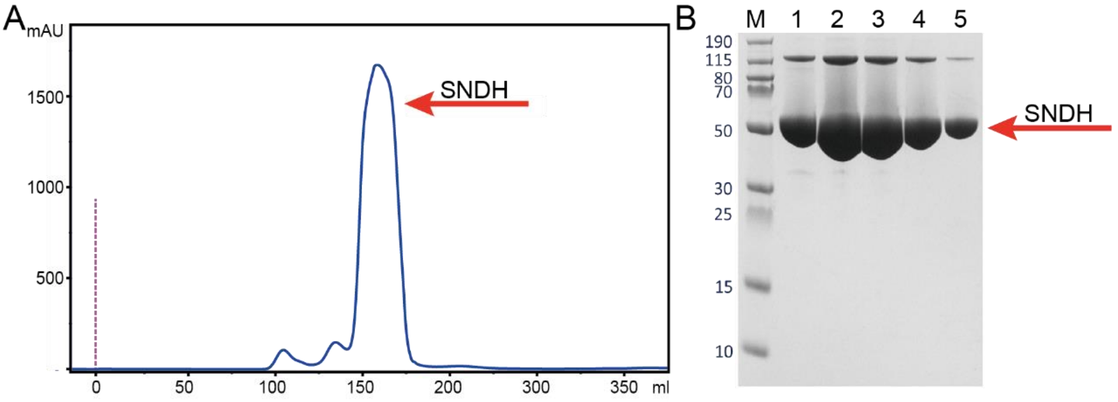

Fig. S1 Size-exclusion chromatography analysis and SDS-PAGE analysis of SNDH.

A: Size-exclusion chromatography data of SNDH; **B:** SDS-PAGE analysis of SNDH. M: marker, 1–5: Collect samples by gel filtration.

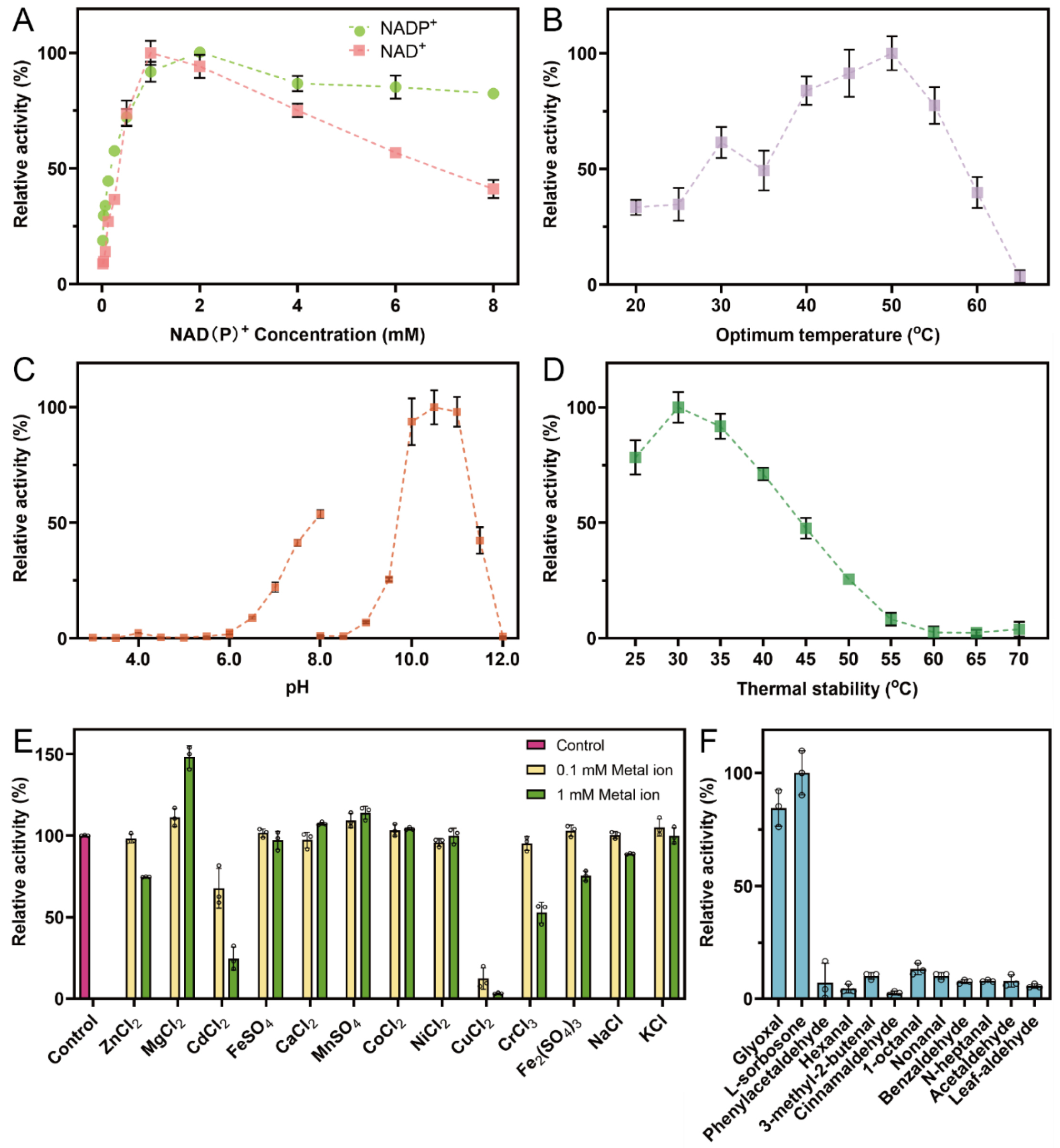

Fig. S2 The enzymatic characteristics of L-sorbosone dehydrogenase.

A: Effect of $NADP^+$ and NAD^+ on SNDH; **B:** Effect of temperature on SNDH; **C:** Effect of pH on SNDH; **D:** Residual activities of SNDH after incubation at different temperature; **E:** Effect of different metal ions on SNDH; **F:** Substrate specificity of SNDH.

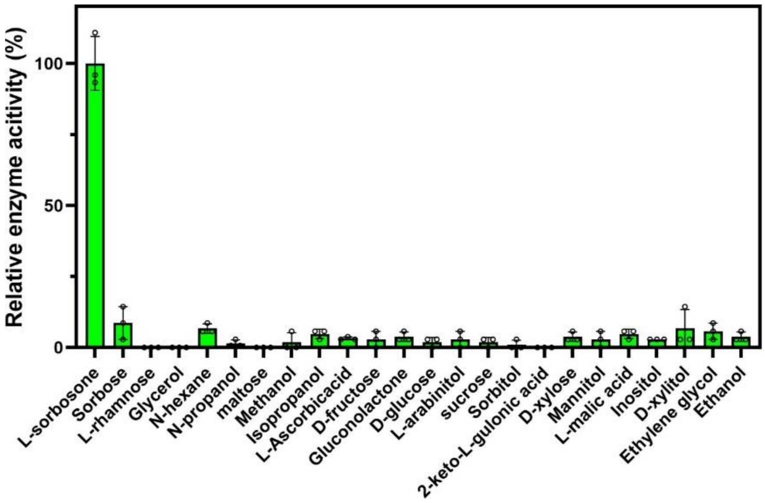

Fig. S3 The alcohols tested for SNDH.

Using a variety of alcohols to test SNDH substrate specificity, the results show that SNDH has low activity on the tested compounds.

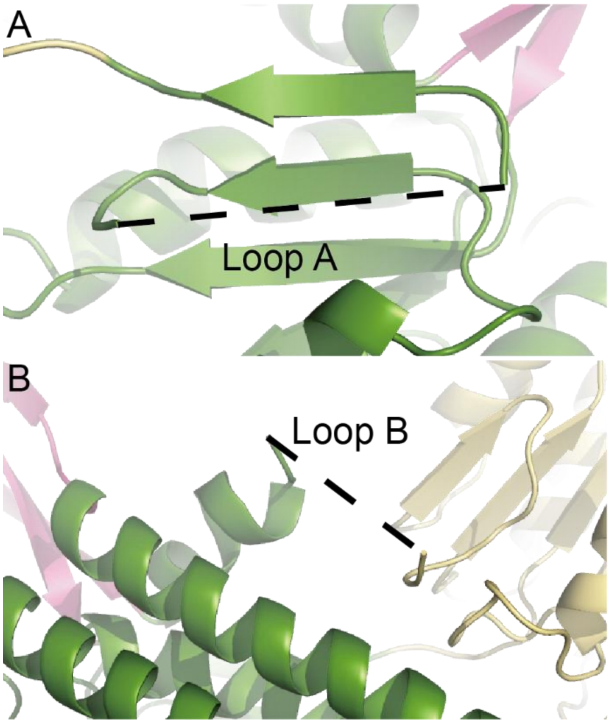

Fig. S4 The missing peptides in SNDH

A: The missing peptides of loop A (Thr241–Asn255); **B:** The missing peptides of loop B (Glu457–Trp468).

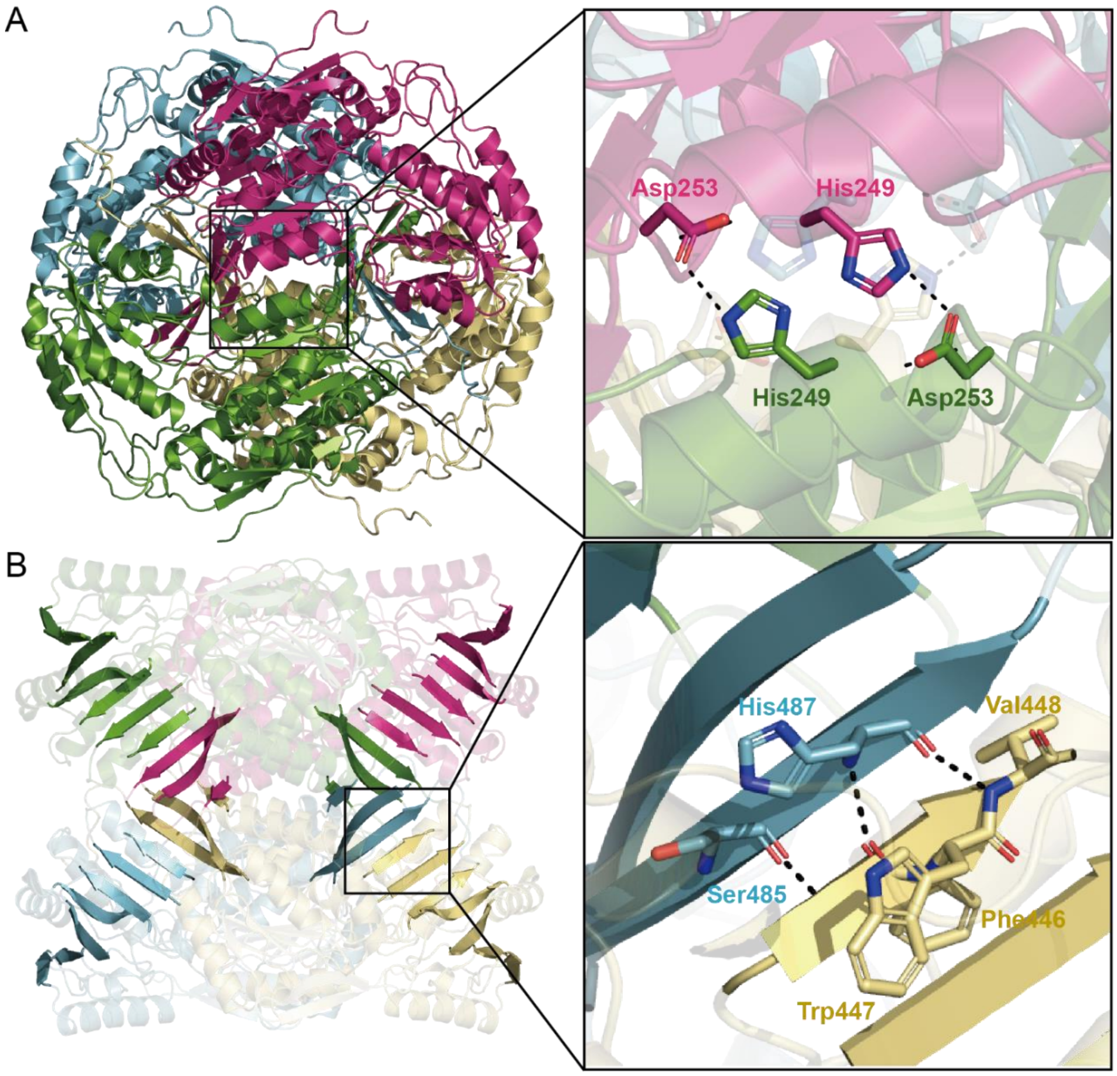

Fig. S5 The overall structural of R-SNDH.

A: The tetrameric structure of mutant R-SNDH. Display the homotetramer of C296A and the interaction between alpha-helix in the subunits. **B:** Display the 10 beta-sheets of R-SNDH and the interaction between beta-sheets in the subunits. Different colors are used to distinguish different subunits. All stick models are colored with elements. The figs are generated by PyMOL.

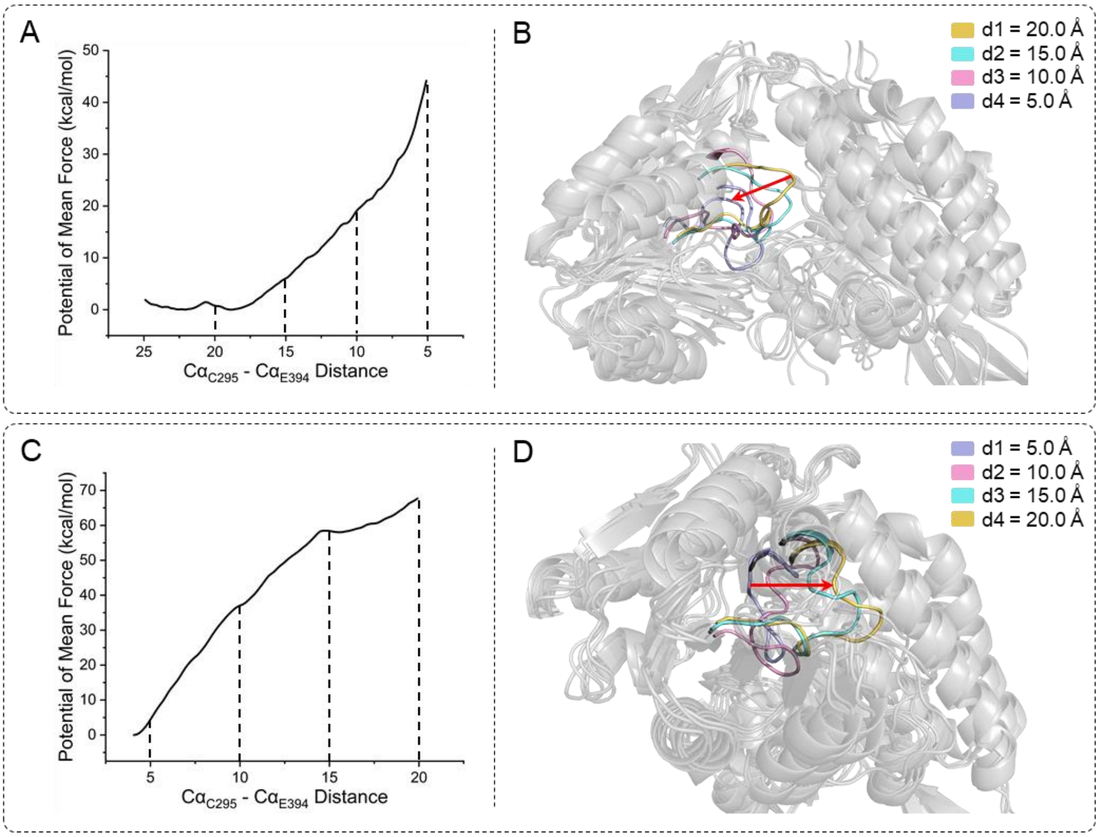

Fig. S6 The closing of O-SNDH and opening of R-SNDH in MD simulations.

A: The potential of mean force in the closing process of O-SNDH. **B:** The structural overlap between the snapshot at the coordinate of 20.0 Å, 15.0 Å, 10.0 Å and 5.0 Å in the closing process of O-SNDH. **C:** The potential of mean force in the opening process of R-SNDH. **D:** The structural overlap between the snapshot at the coordinate of 5.0 Å, 10.0 Å, 15.0 Å and 20.0 Å in the opening process of R-SNDH.

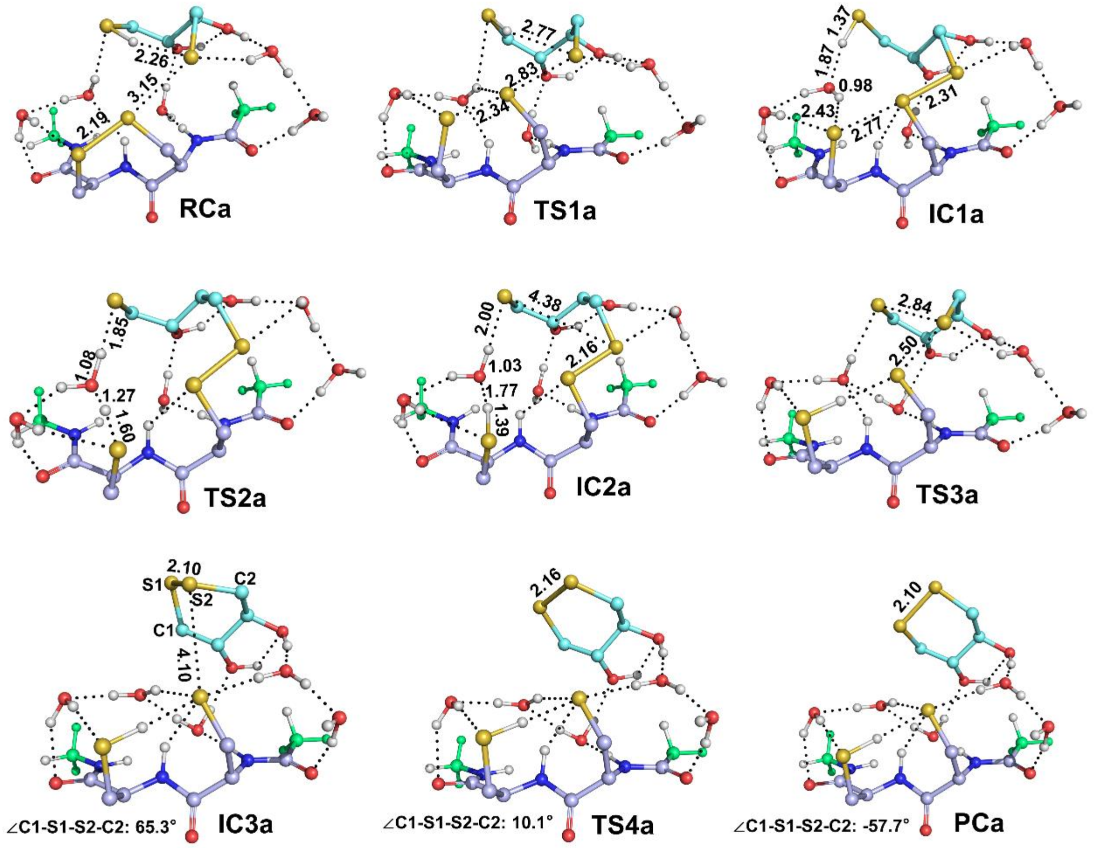

Fig. S7 The geometries in the cleavage processes of disulfide bond mediated by DTT.

The green atoms are kept fixed in geometry optimizations. Key distances are given in Å.

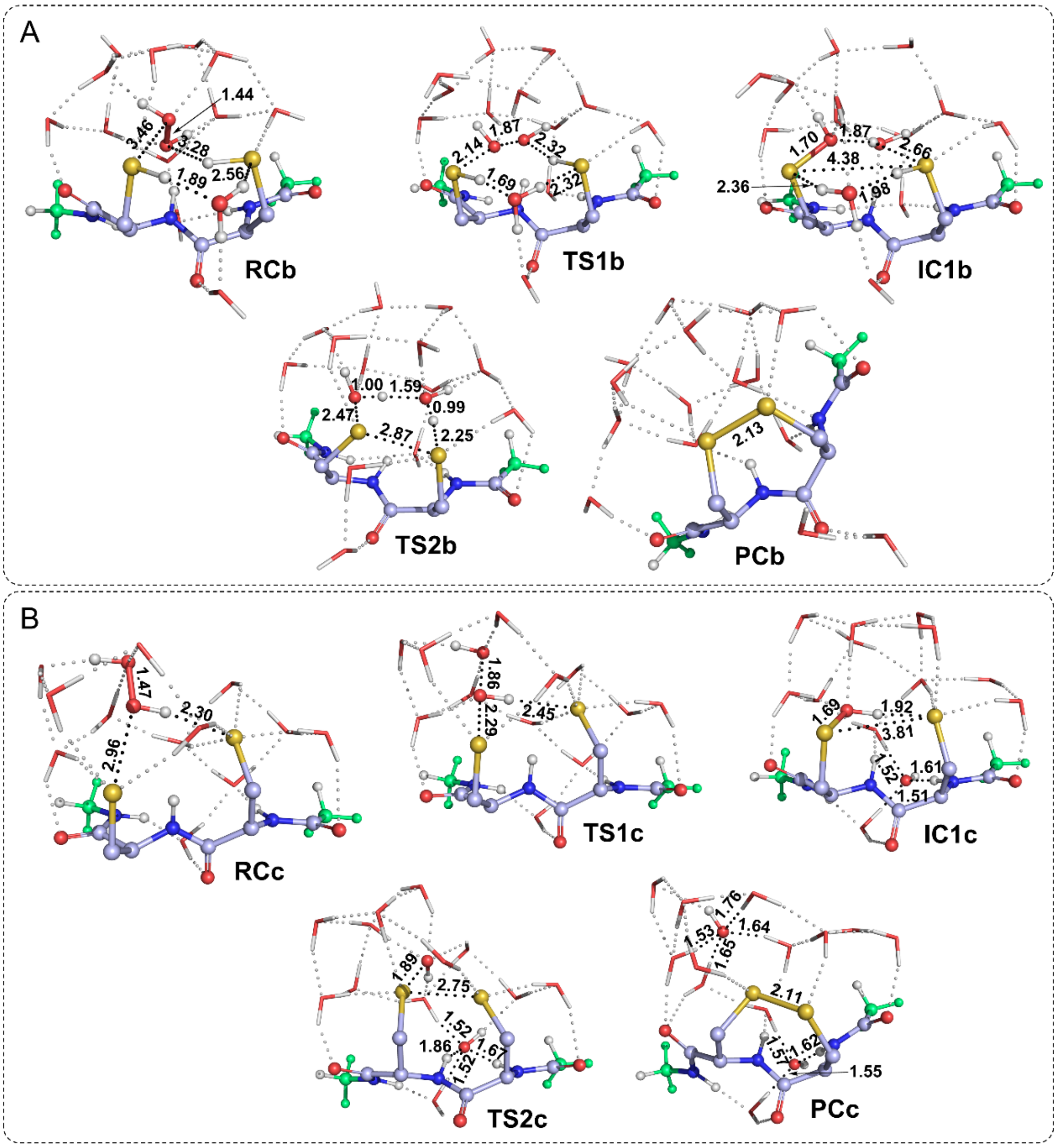

Fig. S8 The geometries in the formation processes of disulfide bond mediated by H_2O_2.

A: The geometries in the formation processes of disulfide bond between protonated Cys295 and Cys296. **B:** The geometries in the formation processes of disulfide bond between deprotonated Cys295 and Cys296. The green atoms are kept fixed in geometry optimizations. Key distances are given in Å.

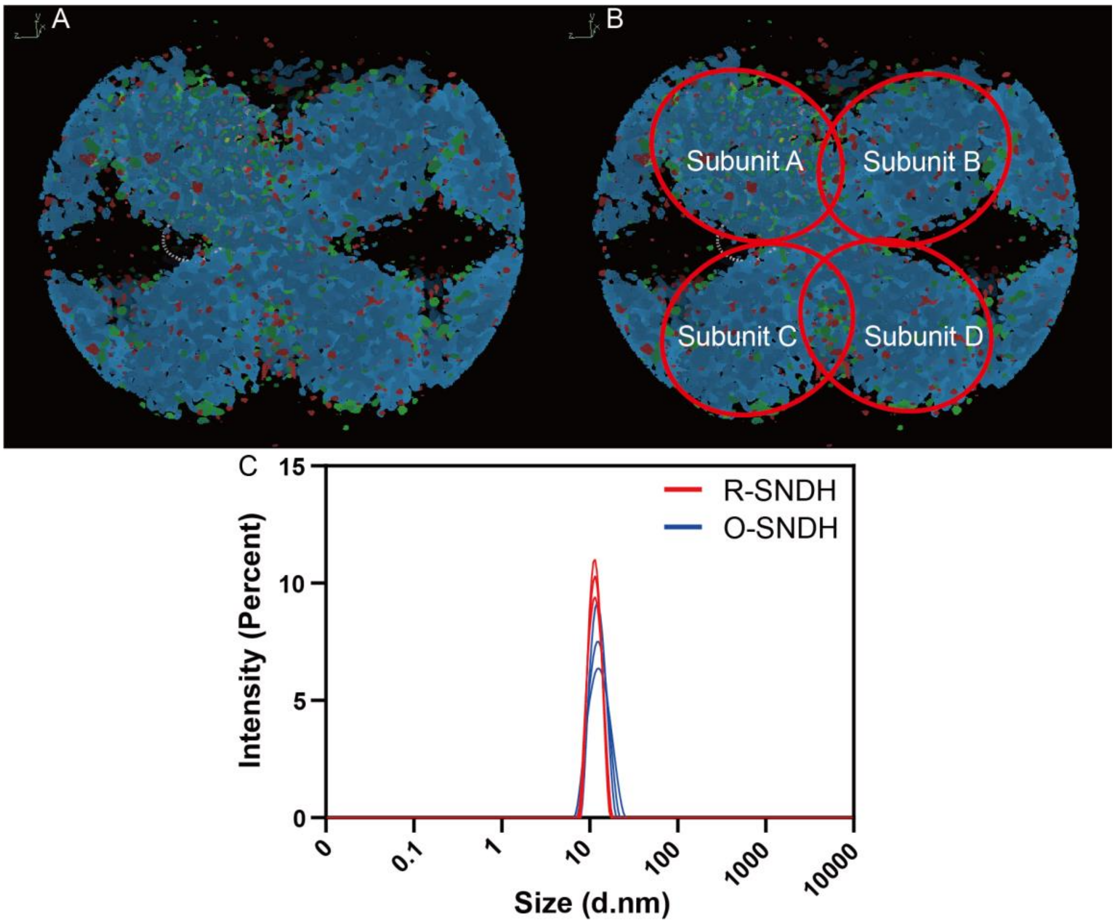

Fig. S9 Electronic density display of O-SNDH

A: Electronic density diagram of O-SNDH. B: Electron density diagram of O-SNDH with subunit position marked. C: The average protein size of R-SNDH and O-SNDH. Observe the electron density diagram with WinCoot, and the Map Radius x-ray value is 60.

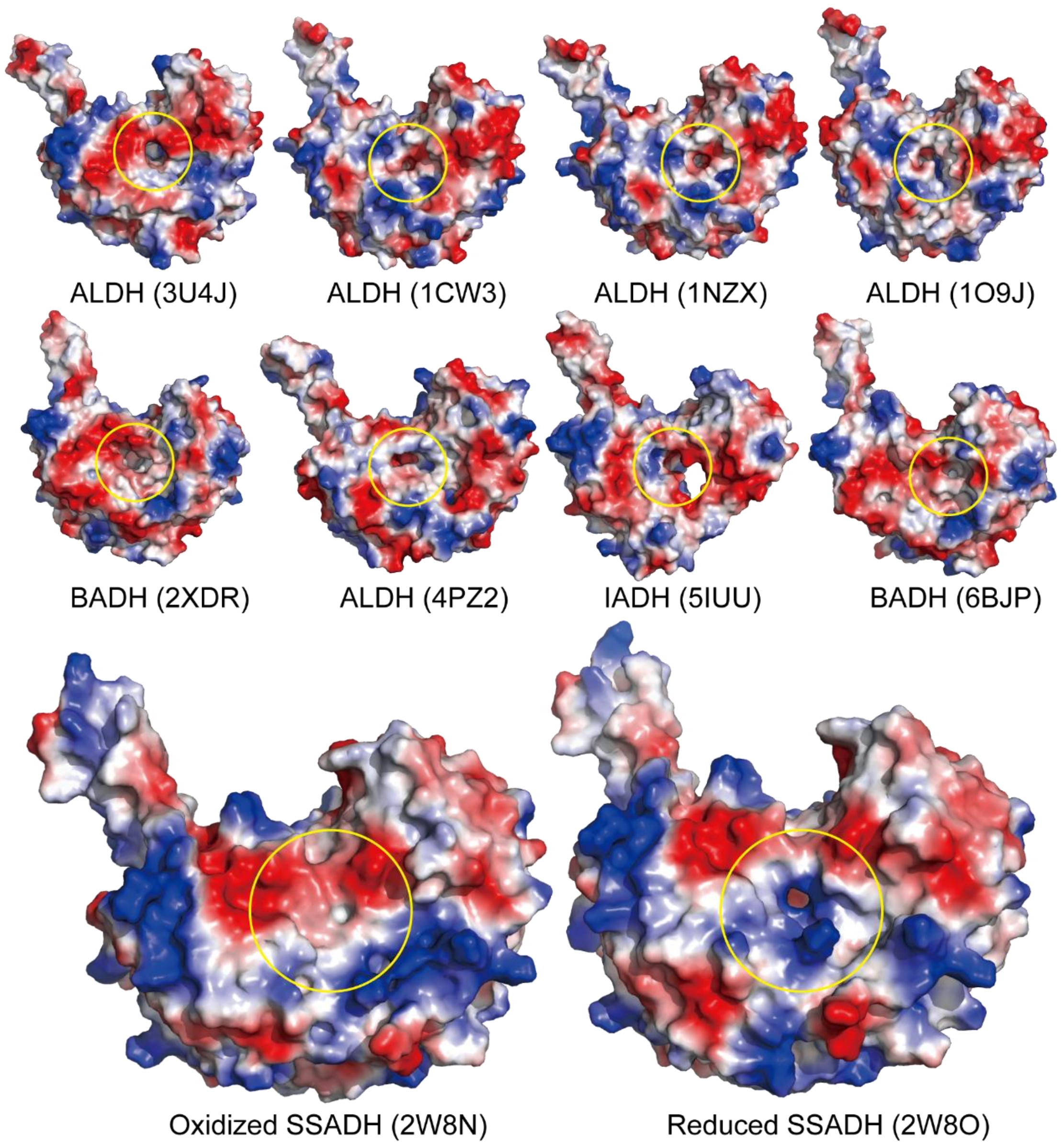

Fig. S10 The overall structural of ALDHs.

ALDH (3U4J), ALDH (1CW3), ALDH (1NZX), ALDH (1O9J), BADH (2XDR), ALDH (4PZ2), IADH (5IUU) and BADH (6BJP) are the members of ALDH-superfamily with an open substrate pocket. SSADH from *Homo sapiens* has an oxidize form and a reduced from, which have a closed substrate pocket (Oxidized SSADH (2W8N)) and an open substrate pocket (Reduced SSADH (2W8O)), respectively. The substrate pockets are marked by yellow circles.

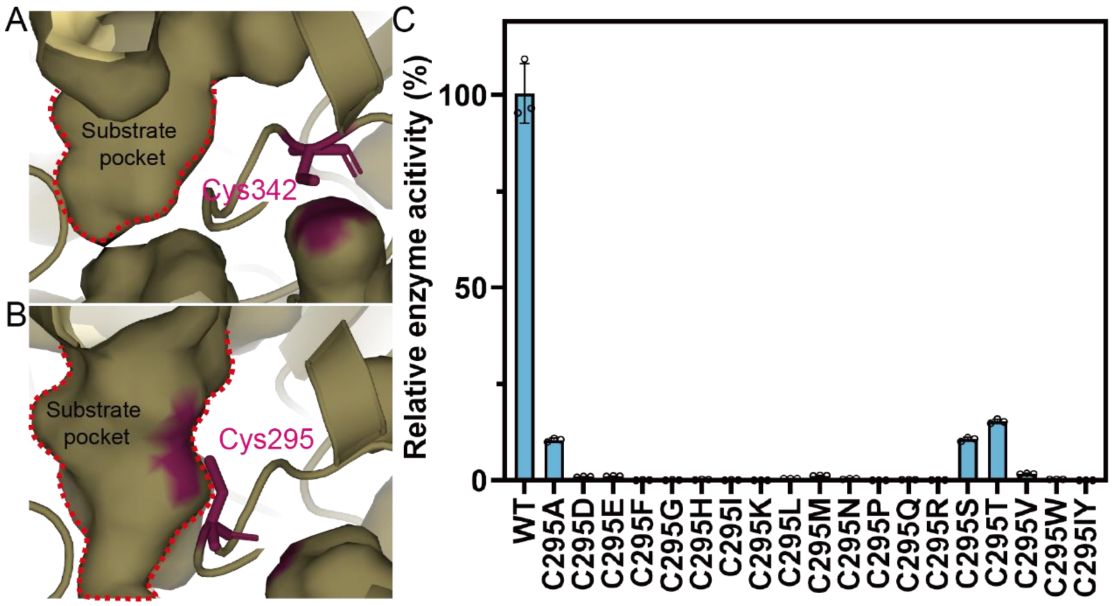

Fig. S11 Substrate pockets differences between SNDH and SSADH.

A: The substrate pocket characterization of SSADH. The structure shows that Cys342 is not the residue that constitutes the substrate pocket; **B:** The substrate pocket characterization of SNDH. The structure shows that Cys295 is part of the substrate pocket. **C:** Effect on SNDH enzymatic activity with Cys295 mutation.

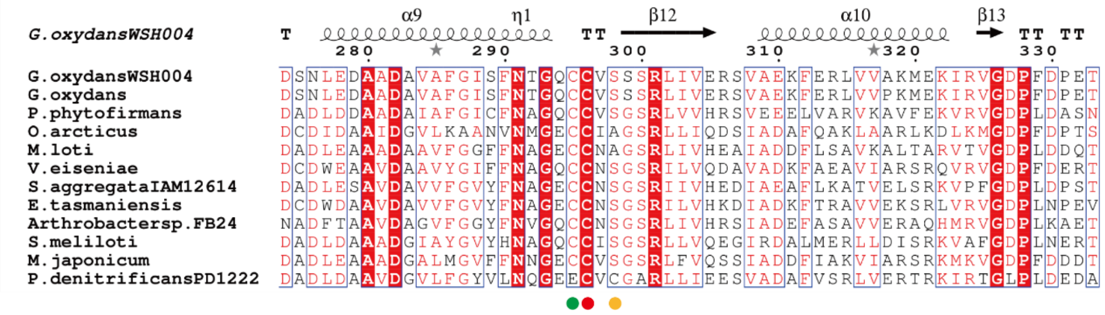

Fig. S12 Multiple sequence alignment of the members of SNDH subfamily.

The red circle marks the position of the catalytic residues Cys of the members in the SNDH-subfamily; the green circle marks the position of non-catalytic residues Cys of the members in the SNDH-subfamily; the yellow circle marks the position of the non-catalytic residue Cys of *P. denitricans* SNDH.

Supplementary Tables

Table S1 Comparison of SNDH and known structural sequence similarity

PDB code	Organism	Max Score	Query Cover	Per. Ident
3U4J	*Sinorhizobium meliloti*	424	93%	47.44%
2WME	*Pseudomonas aeruginosa*	370	92%	42.24%
1O9J[55]	*Elephantulus edwardii*	370	96%	41.72%
6BJP	*Pseudomonas aeruginosa*	369	92%	42.24%
4PZ2[56]	*Zea mays*	369	93%	44.04%
2XDR	*Pseudomonas aeruginosa*	367	92%	42.03%
1CW3[57]	*Homo sapiens*	367	95%	40.71%
5IUU[58]	*Pseudomonas syringae*	367	94%	42.11%
1NZX[59]	*Homo sapiens*	367	95%	40.71%
2WOX[60]	*Pseudomonas aeruginosa*	367	92%	42.03%

Table S2 Primers for the construction of plasmid pET-SNDH

**Name***	**Primers (5'-3')**
SNDH-F	AACTTTAAGAAGGAGATATACCATGAATGTTGTCTCAAAGACTGTATCTTTACCG
SNDH-R	GGCTTTGTTAGCAGCCGGATCTTACGAAATCCAGTGCGAACGTTTG
pET28a-F	GATCCGGCTGCTAACAAAGCCCGAAAG
pET28a-R	GGTATATCTCCTTCTTAAAGTTAAACAAAATTATTTCTAGAGGGG

*: SNDH-F and SNDH-R are used to clone SNDH. pET28a-F and pET28a-R are used to linearize plasmid pET-28a(+).

Table S3 Primers for the construction of SNDH mutant

Name	Primers (5'-3')
N163A-F	ACGCCGTGGgcgTTCCCGTTCATGATCCTGTGTGAGCGG
N163A-R	AACGGGAAcgcCCACGGCGTAATCAGACCGACGA
R172A-F	TGTGAGgcgGCGCCTTTCATTCTCGCATCC
R172A-R	AGGCGCcgcCTCACACAGGATCATGAACGGGAA
K186A-F	CTGGTCGTCgcgCCTGCCGAAGTCACGAGTGCC
K186A-R	TTCGGCAGGcgcGACGACCAGCGTGCAGCC
E262A-F	GGCCTCgcgCTGGGCGGCAAGAACCC
E262A-R	GCCCAGcgcGAGGCCAAGTTTCTTCAGGTTGC
G293A-F	AATACCgcgCAGTGCTGTGTGTCGTCGAG
G293A-R	GCACTGcgcGGTATTAAAGCTGATCCCGAAGGCT
C295A-F	TACCGGGCAGgcgTGTGTGTCGTCGAGCCGCCTG
C295A-R	CGACACACAcgcCTGCCCGGTATTAAAGCTGATCCCG
C295D-F	TACCGGGCAGgatTGTGTGTCGTCGAGCCGCCTG
C295D-R	CGACACACAatcCTGCCCGGTATTAAAGCTGATCCCG
C295E-F	TACCGGGCAGgagTGTGTGTCGTCGAGCCGCCTG
C295E-R	CGACACACActcCTGCCCGGTATTAAAGCTGATCCCG
C295F-F	TACCGGGCAGttcTGTGTGTCGTCGAGCCGCCTG
C295F-R	CGACACACAgaaCTGCCCGGTATTAAAGCTGATCCCG
C295G-F	TACCGGGCAGgggTGTGTGTCGTCGAGCCGCCTG
C295G-R	CGACACACAcccCTGCCCGGTATTAAAGCTGATCCCG
C295H-F	TACCGGGCAGcatTGTGTGTCGTCGAGCCGCCTG
C295H-R	CGACACACAatgCTGCCCGGTATTAAAGCTGATCCCG

C295I-F	TACCGGGCAGatcTGTGTGTCGTCGAGCCGCCTG
C295I-R	CGACACACAgatCTGCCCGGTATTAAAGCTGATCCCG
C295K-F	TACCGGGCAGaagTGTGTGTCGTCGAGCCGCCTG
C295K-R	CGACACACActtCTGCCCGGTATTAAAGCTGATCCCG
C295L-F	TACCGGGCAGcttTGTGTGTCGTCGAGCCGCCTG
C295L-R	CGACACACAaagCTGCCCGGTATTAAAGCTGATCCCG
C295M-F	TACCGGGCAGatgTGTGTGTCGTCGAGCCGCCTG
C295M-R	CGACACACAcatCTGCCCGGTATTAAAGCTGATCCCG
C295N-F	TACCGGGCAGaatTGTGTGTCGTCGAGCCGCCTG
C295N-R	CGACACACAattCTGCCCGGTATTAAAGCTGATCCCG
C295P-F	TACCGGGCAGcccTGTGTGTCGTCGAGCCGCCTG
C295P-R	CGACACACAgggCTGCCCGGTATTAAAGCTGATCCCG
C295Q-F	TACCGGGCAGcagTGTGTGTCGTCGAGCCGCCTG
C295Q-R	CGACACACActgCTGCCCGGTATTAAAGCTGATCCCG
C295R-F	TACCGGGCAGcgcTGTGTGTCGTCGAGCCGCCTG
C295R-R	CGACACACAgcgCTGCCCGGTATTAAAGCTGATCCCG
C295S-F	TACCGGGCAGtcgTGTGTGTCGTCGAGCCGCCTG
C295S-R	CGACACACAcgaCTGCCCGGTATTAAAGCTGATCCCG
C295T-F	TACCGGGCAGaccTGTGTGTCGTCGAGCCGCCTG
C295T-R	CGACACACAggtCTGCCCGGTATTAAAGCTGATCCCG
C295V-F	TACCGGGCAGgtaTGTGTGTCGTCGAGCCGCCTG
C295V-R	CGACACACAtacCTGCCCGGTATTAAAGCTGATCCCG
C295W-F	TACCGGGCAGtggTGTGTGTCGTCGAGCCGCCTG
C295W-R	CGACACACAccaCTGCCCGGTATTAAAGCTGATCCCG
C295Y-F	TACCGGGCAGtacTGTGTGTCGTCGAGCCGCCTG

C295Y-R	CGACACACAgatCTGCCCGGTATTAAAGCTGATCCCG
C296A-F	GGGCAGTGCgcgGTGTCGTCGAGCCGCCTGATC
C296A-R	GACGACACcgcGCACTGCCCGGTATTAAAGCTGATCC C
E394A-F	GCGCGTGACgcgATTTTTGGGCCGGTTCTGGCGTC
E394A-R	CCCAAAAATcgcGTCACGCGCGATGCCC
E471D-F	GCCGTgacGCCGGTCTGTACGGCGTTGAGGAATA
E471D-R	CGGCgtcACGGCCCCAGCCCGAC

*: Lowercase letters in primers represent mutation sites.

Table S4 Data collection and refinement statistics.

Parameters	Oxidized SNDH	Reduced SNDH	C296A
Resolution range (Å)	29.07 - 2.504 (2.593 - 2.504)	23.5 - 2.988 (3.095 - 2.988)	26.66 - 2.218 (2.297 - 2.218)
Space group	P 6222	P 1 21 1	P 1 21 1
Unit cell			
a, b, c (Å)	181.018, 181.018, 77.593	80.242, 118.817, 109.02	80.305, 117.928, 108.179
α, β, γ (°)	90, 90, 120	90 93.319 90	90 95.138 90
Completeness (%)	99.25 (99.26)	99.52 (97.91)	92.65 (66.47)
Wilson B-factor (Å^2)	36.1	22.89	32.08
Reflections used in refinement	26077 (2547)	41280 (4021)	92006 (6578)
Reflections used for R-free	1229 (133)	1976 (172)	4712 (327)
*R*work	0.1977 (0.2600)	0.1723 (0.2272)	0.2028 (0.2613)
*R*free	0.2393 (0.3099)	0.2368 (0.3126)	0.2369 (0.3387)
Number of non-hydrogen atoms	3599	14886	14865
Macromolecules	3464	14694	14673
RMS bonds (Å)	0.009	0.012	0.01
RMS angles (°)	1.27	1.37	1.31

Ramachandran plot (%)			
Favored	95.12	95.97	96.38
Allowed	4.66	3.77	3.41
Outliers	0.22	0.26	0.21
Rotamer outliers (%)	0	0.53	0.27
Clashscore	8.23	11.2	10.96
Average B-factor (Å^2)	39.17	19.55	34.52
Macromolecules	39.07	19.15	34.48
PDB code	7W5L	7W5N	7W5K
